# Twenty years of herpes simplex virus type 2 (HSV-2) research in low-income and middle-income countries: systematic evaluation of progress made in addressing WHO prioritiesfor research in HSV-2 epidemiology and diagnostics

**DOI:** 10.1136/bmjgh-2023-012717

**Published:** 2024-07-04

**Authors:** Muna Jama, Ela Mair Owen, Belinder Nahal, Angela Obasi, Emily Clarke

**Affiliations:** 1 Liverpool School of Tropical Medicine, Liverpool, UK; 2 International Rescue Committee, Mogadishu, Somalia; 3 University of Liverpool, Liverpool, UK; 4 London School of Hygiene & Tropical Medicine, London, UK; 5 Department of International Public Health, Liverpool School of Tropical Medicine, Liverpool, UK; 6 Axess Sexual Health, Liverpool University Hospitals NHS Foundation Trust, Liverpool, UK

**Keywords:** Epidemiology, Diagnostics and tools, Public Health

## Abstract

**Introduction:**

Low-income and middle-income countries (LMICs) have a high burden of herpes simplex virus type 2 (HSV-2) infection, which has been strongly associated with HIV. In 2001, the WHO hosted a workshop to set research priorities for HSV-2 in LMICs. Periodic re-evaluation of research priorities is essential to ensure effective allocation of resources. This study describes the progress made between 2000 and 2020 in addressing the priorities identified in two of the five thematic areas that were the workshop’s focus: HSV-2 epidemiology and diagnostics. The remaining areas are addressed in a companion paper.

**Methods:**

A systematic search of MEDLINE, CINAHL, Global Health and Cochrane databases was carried out. Relevant primary and secondary research studies conducted in LMICs, written in English and published from 2000–2020 were included. Two independent researchers screened, identified papers and extracted preidentified variables from study texts. Data were organised into an Excel spreadsheet and analysed using IBM SPSS V.26.

**Results:**

Overall, 4445 discrete papers were identified, of which 165 publications were eligible for inclusion. The highest general population HSV-2 prevalence was reported in South and West Africa. Prevalence was higher among women than men and increased with age. HSV-2 prevalence studies among key populations were few, and the majority were in East and South Asia. Cohort studies of HSV-2 incidence among younger populations (mean age=25 years) and HSV-2 infection prevalence in North Africa and the Middle East were few. The most researched topic in HSV-2 diagnostics addressed serological techniques and direct molecular biology. Studies of point-of-care testing were also few.

**Conclusion:**

HSV-2 research identified in LMICs has mainly addressed the epidemiology and diagnostics priorities identified by the 2001 WHO workshop. Unaddressed priorities include point-of-care testing, antiviral resistance and exploration of HSV-2 epidemiology in neglected geographical settings and population subgroups.

**WHAT IS ALREADY KNOWN ON THIS TOPIC**

- Herpes simplex virus type 2 (HSV-2) is a sexually transmitted infection, which affects over 491.5 million adults globally with the highest rates being found in low-income and middle-income countries (LMICs). In 2001, the WHO held an international workshop to set future HSV-2 research priorities for LMICs. Priority setting is important for optimising research resource allocation. Review of research progress against identified priorities is important to ensuring continuing research relevance but is rarely done. This study aims to review the progress made in HSV-2 epidemiology and HSV-2 diagnostics research in LMICs from 2000 to 2020.

**WHAT THIS STUDY ADDS**

- Although most HSV-2 research priorities identified by the 2001 WHO workshop in HSV-2 epidemiology and HSV-2 diagnostics for LMICs have been addressed, knowledge gaps remain in point-of-care testing, exploration of HSV-2 epidemiology in neglected geographical settings such as North Africa, the Middle East and various population subgroups.

**HOW THIS STUDY MIGHT AFFECT RESEARCH, PRACTICE OR POLICY**

- Gaps and unaddressed priorities that we have identified can inform further priority setting and research programming for HSV-2 research in LMICs.

## Introduction

Herpes simplex virus type 2 (HSV-2) is the primary cause of genital herpes infection globally and is almost exclusively sexually transmitted.^1 2^ It is a lifelong, incurable infection affecting nearly half a billion people aged 15–49 and is considered of major public health significance.^2^ The infection is highly transmissible and may be associated with lifelong recurrence of symptoms, including severe pain, and is the predominant cause of genital ulceration worldwide.^3^ Serious complications include neonatal infection and meningitis.^4 5^ HSV-2 has been associated with the acquisition and transmission of HIV in HIV epidemic regions.^6^ Increasing evidence has found that persons with HSV-2 seropositive are up to five times more likely to acquire HIV, and persons living with HIV who become HSV-2 seropositive are more likely to transfer the virus to their sexual partners.^7^

The highest burden of HSV-2 has been reported in low-income and middle-income countries (LMICs), with women (313.5 million) being infected at a higher rate than men (178.0 million).^2^ Populations in Africa have the highest HSV-2 prevalence, followed by the Western Pacific, South-East Asia and Americas regions.^2^ There is an increased incidence of HSV-2 among populations with higher-risk sexual behaviours, for example, men having sex with other men (MSM) and female sex workers (FSW) compared with the general population.^8 9^

In 2001, in response to the substantial burden of HSV-2 and a growing body of evidence linking HSV-2 to HIV prevalence and incidence, the WHO hosted a 3-day workshop to set research priorities for HSV-2 in LMICs.^10^ Setting health research priorities is essential for optimised use of research resources.^11 12^ This is particularly important for resource-poor countries. Priorities are best developed through the application of defined methodologies by multiple stakeholders, including researchers, policy-makers, healthcare providers and service users.^13^ The workshop brought together a broad range of regional and disciplinary expertise. The participants were from Europe (n=18), Africa (n=10), North America (n=7) and Asia (n=1). Their expertise spanned areas of biochemistry, clinical medicine, programme interventions and mathematical disease modelling, and most of them represented well-established global health research organisations and educational institutions. Most of the organisations present originated from high-income countries (HIC), such as the USA (6 institutes) and the UK (14 organisations and educational institutions). Eight institutions were based in LMICs: Zimbabwe (n=3), Uganda (n=3), South Africa (n=1) and India (n=1) (further described in companion paper^14^). The workshop included expert presentations, plenary dialogues and breakout discussion groups. On the final day, a list of recommendations made during the discussions was examined and prioritised in five key areas: HSV-2 epidemiology, HSV-2 diagnostic, HSV-2/HIV interactions, HSV-2 control measures and HSV-2 mathematical modelling.

In the absence of periodic re-evaluation of research priorities, there is a risk that research conducted will fail to respond to actual health needs or promote effective allocation of resources.^15^ To our knowledge, no review has been published to examine the progress made towards the research priorities defined in the 2001 WHO workshop.

This review aims to assess progress in addressing research priorities in two of the five areas, namely: HSV-2 epidemiology and HSV-2 diagnostics. The remaining three research priorities (HSV-2/HIV interactions, HSV-2 control measures and HSV-2 mathematical modelling) are addressed in a companion paper.^16^

Table 1 summarises subthemes identified in the 2001 WHO workshop in the two areas that are the subject of the current review.

**Table 1:** Research priorities set in the 2001 WHO HSV-2 workshop

HSV-2 epidemiology	HSV-2 diagnostics
HSV-2 prevalence in South America, Africa, and Asia. Genital ulcer disease aetiology, particularly in STI patients by HIV status and sex. The emergence of aciclovir resistance status in LMICs.	Optimal sampling techniques for direct diagnostic methods. Quantification techniques for HSV-2 DNA detection. Development of a rapid diagnostic test for genital herpes diagnosis. Performance of existing serological tests in different African sera.

HSV-2, herpes simplex virus type 2; LMICs, low-income and middle-income countries; STI, sexually transmitted infection.

## Methods

**Figure 1:**
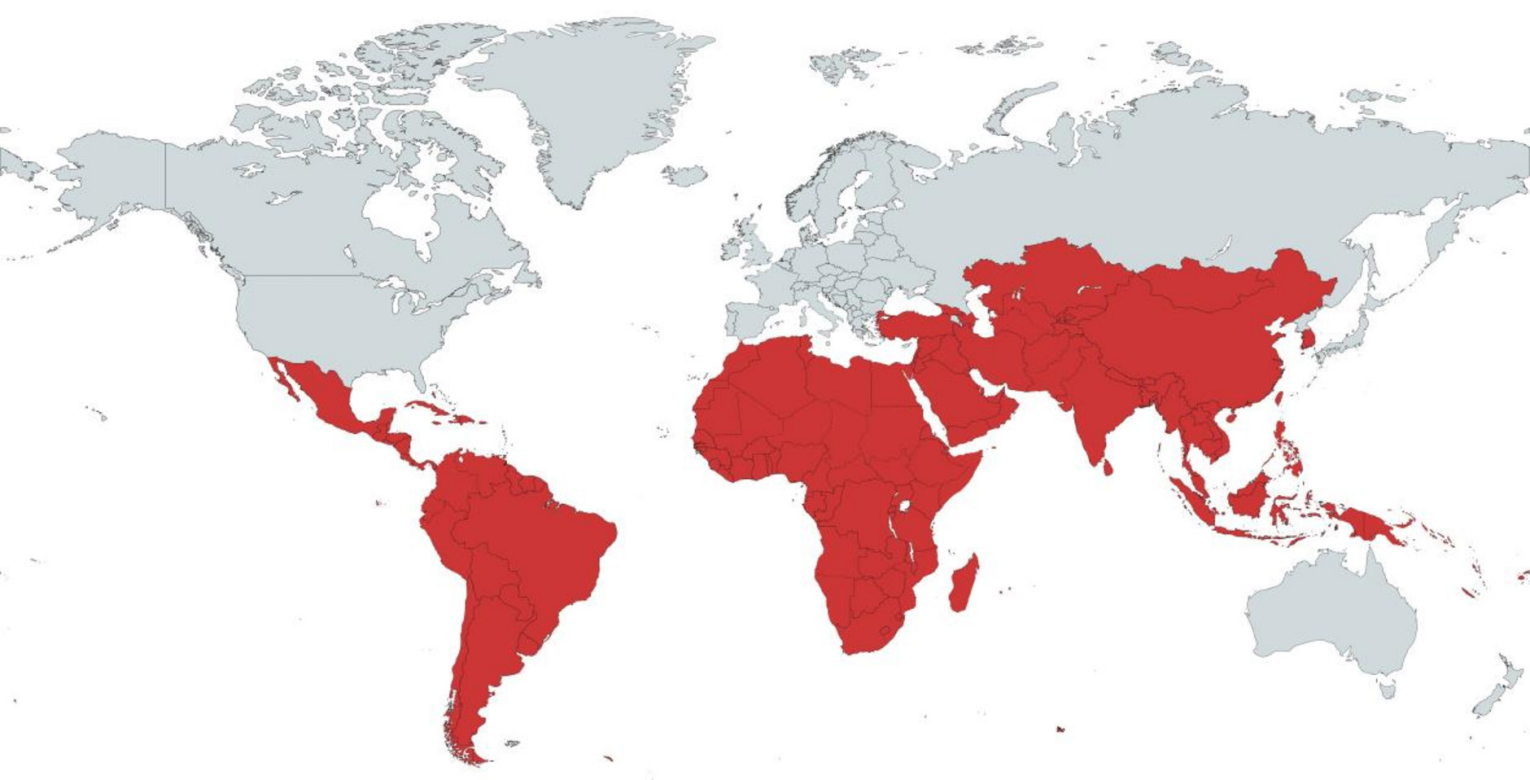
Map displays in red the LMICs that were eligible for inclusion in this study based on the United Nations classification by income and development. LMICs, low-income and middle-income countries.

A systematic scoping of the literature was conducted to review progress between 2000 and 2020 in HSV-2 epidemiology and HSV-2 diagnostics. The detailed methods are presented in a companion paper.^16^ However, specific search terms were selected for each priority area, ‘low and middle-income countries’ and ‘HSV-2. Boolean operators ‘OR’ and ‘AND’ were used to combine the search terms (online supplemental appendix 1). Searches were conducted on MEDLINE, CINAHL, Global Health and Cochrane databases. Primary and secondary research conducted in LMICs published in English between 2000 and 2020 were included. Two independent reviewers screened identified records and assessed them for quality using preidentified quality indicators^16^ before undergoing detailed content analysis. Studies were allocated as HIC/LMIC according to the UN country classification system.^17^ The map in figure 1 shows the countries eligible for inclusion. Data were charted using Microsoft Excel V.365 and BM SPSS V.26.

10.1136/bmjgh-2023-012717.supp1 Supplementary data

### Patient and public involvement

Patient and public involvement was not appropriate for this study as no new patient data were collected.

## Results

### Literature search

**Figure 2:**
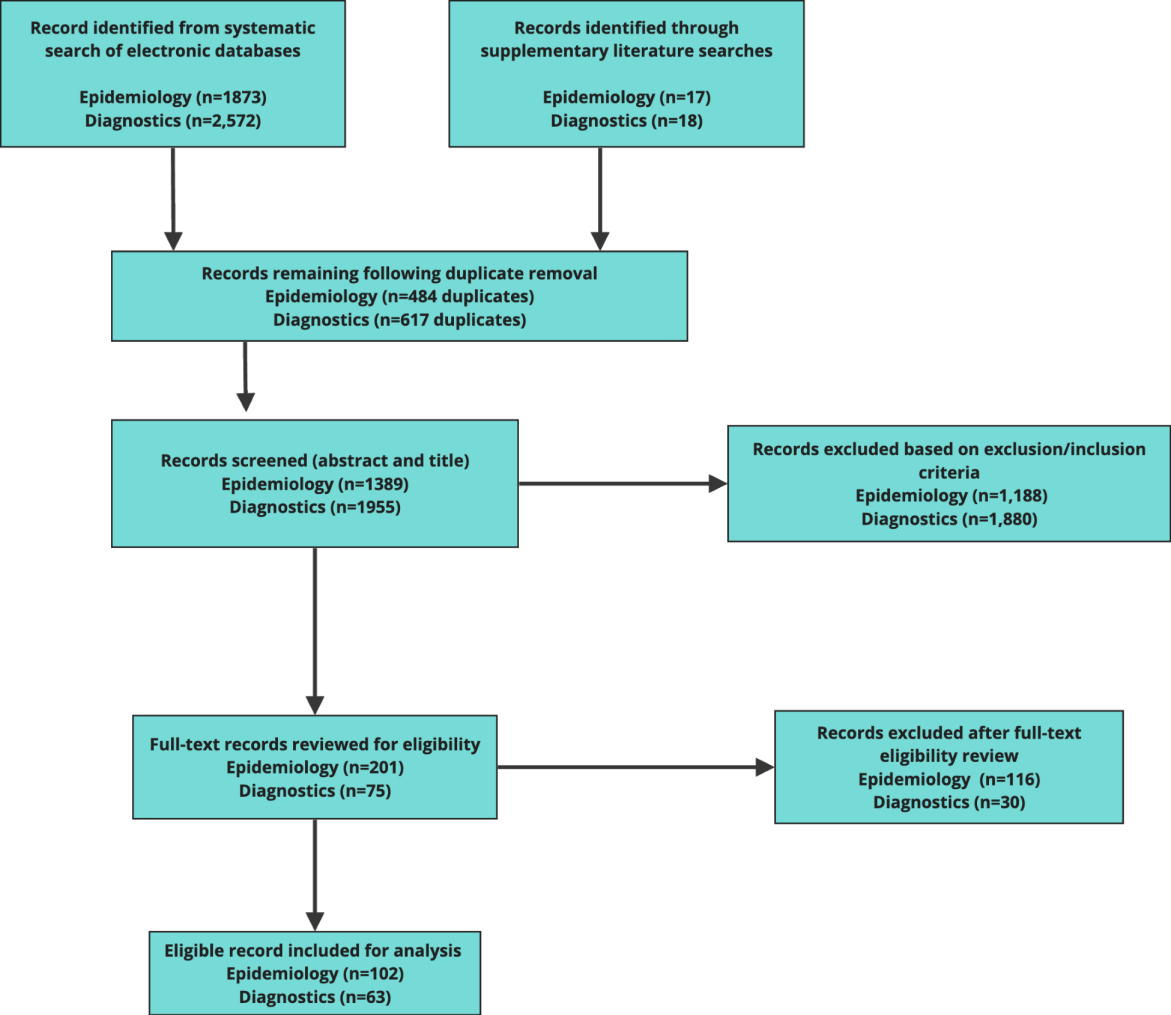
The study selection process for HSV-2 epidemiology and HSV-2 diagnostic. Online supplemental appendix 3 shows the reasons for exclusion following a full-text eligibility review. HSV-2, herpes simplex virus type 2.

A total of 4445 publications were identified: 1873 (HSV-2 epidemiology) and 2572 (HSV-2 diagnostics). Removal of 1101 duplicates left 3344 publications. The abstract and title of each were examined for potential relevance, resulting in exclusion of 3086 records based on the inclusion and exclusion criteria.^16^ This left 258 papers for which the full text was obtained. An additional 35 potentially relevant publications were identified through reference lists. After full-text retrieval and reviewal, 128 publications were excluded, leaving 165 (HSV-2 epidemiology=102, HSV-2 diagnostics=63) publications included for the content analysis and data extraction. For the two searches, there was a high level of screening concordance (>99%) between reviewers. A full list of the included studies can be viewed in online supplemental appendix 2. Figure 2 provides a flow diagram of the literature selection process for the two research areas.

10.1136/bmjgh-2023-012717.supp2 Supplementary data

10.1136/bmjgh-2023-012717.supp3 Supplementary data

### HSV- 2 epidemiology

A total of 102 relevant studies reporting on HSV-2 epidemiology were identified between 2000 and 2020. The studies included in this review covered LMICs in almost all world regions. The most common study locations were East Asia 19 (18.6%), followed by South Asia 15 (14.7%), East Africa 14 (13.7%) and South America 11 (10.8%). A total of 10 studies were based in West Africa and Southern Africa, respectively (9.8%). Nine were in Central America (8.8%), three were in the Caribbean and Central Africa, respectively (2.9%) and eight (7.8%) studies were based across multiple regions. No study in the Middle East, North Africa and Western Asia regions was identified. Regarding the most commonly studied topic, 51 (50%) studies identified HSV-2 seroprevalence among the general population, defined as those with no specific higher-risk sexual practices.24 (23.5%) studies of HSV-2 seroprevalence were conducted in groups who, due to specific higher-risk sexual practices, are at an increased risk of acquiring HSV-2 infection, such as FSW and MSM.^7^

Of the 102 articles reviewed, 36 (35.3%) studies aimed to estimate the HSV-2 prevalence among the female population, 15 (14.7%) in male participants and 46 (45.1%) assessed both population groups, while 5 (4.9%) studies estimated HSV-2 prevalence by conducting systematic reviews. Among the 102 selected articles, 16 (15.6%) studies estimated HSV-2 prevalence among adolescents. HSV-2 seroprevalence estimates were examined using specific types of ELISA test kits 74 (72.5%), syndromic diagnosis17 (16.6 %) and PCR 11 (10.7 %). 57 (55.9%) articles examined HSV-2 in a community-based setting,33 (32.4%) in a hospital or STI clinical setting and 12 (11.7%) studies investigated HSV-2 in both settings.

#### Prevalent HSV-2 infection in LMICs

The HSV-2 prevalence was higher among women than men and increased with age regardless of geographical location and subgroup (eg, people living with HIV, sex workers and pregnant women).

The highest HSV-2 prevalence estimate was reported among the general population in West Africa (87.0% in individuals aged 11–60 years)^18^ and South Africa regions (65.0% in individuals aged 18–64 years),^19^ although most identified studies were from East Africa. HSV-2 prevalence studies among high-risk populations were few. A total of 24 studies were identified. Most studies were from East and South Asia 15 (63%), followed by East Africa 4 (17%), Central and South America 3 (12%) and West and South Africa regions 2 (8%). Cohort studies of HSV-2 incidence among younger populations (mean age=25 years) were limited. Only four cohort studies were identified in the African regions, where high HSV-2 estimates were found among the younger population.

#### HSV-2 as the cause of genital ulcer disease

11 studies examined HSV-2 as a cause of genital ulcer disease (GUD). Most studies were conducted in South America five (45%), with 2 (18%) studies in the East Asia and East Africa regions, respectively. Of the 11 identified studies, HSV-2 was the most common cause of GUD.

Table 2 summarises progress in addressing HSV-2 epidemiology-associated research priorities set in the 2001 WHO HSV-2 workshop.

**Table 2:** HSV-2 epidemiology research progress according to the associated research priorities set in the 2001 WHO HSV-2 workshop

WHO 2001 research priority: HSV-2 epidemiology	Has the priority been addressed?			Explanation
	Yes	Partially	No	
HSV-2 prevalence in LMICs		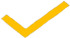		0 studies based in the Middle East, North Africa, Eastern Europe and Western Asia regions. 4 cohort studies of HSV-2 incidence among younger populations (mean age=25 years) in the African regions. HSV-2 prevalence studies among high-risk populations were few, and the majority were in East and South Asia.
HSV-2 as the cause of genital ulcer disease (GUD) aetiology		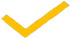		Most studies in South America (n=5) 0 cohort studies to clarify the natural history and transmission of HSV-2. More studies are required to understand contribution of HSV-2 to GUD in LMICs
Aciclovir resistance status in LMICs			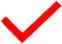	Antiviral resistance, including aciclovir, was also highly understudied. 1 study in total.

HSV-2, herpes simplex virus type 2; LMICs, low-income and middle-income countries.

### HSV- 2 diagnostics

A total of 63 studies assessing HSV-2 diagnostics in LMICs between 2000 and 2020 were identified. The most frequently researched topic was serological diagnostic techniques with 33 (52.3%) studies. Other less frequently studied techniques were molecular biology/DNA detection methods 16 (25.3%), syndromic diagnosis 12 (19.0%) and diagnostic sampling techniques 6 (9.5%). 36 (57.1%) studies included both male and female participants, 16 (25.3%) were included females only and 5 (7.9%) included males only. Geographically, the most studied areas were East Asia 14 (22.2%) and East Africa 12 (19.0%). South Asia and Southern Africa had 11 (17.4%) and 8 (12.6%) studies, respectively, and both West Africa and South America had 7 studies (11.1%) each. Central Africa and Western Asia had five (7.9%) and three (4.7%) studies, respectively, and both Central America and North Africa had one (1.5%) each. Regarding the study population, most studies 26 (41.2%) focused on clinic attendees and/or symptomatic people presumed to have GUD. Other populations studied included the general population 12 (19.0%), commercial sex workers 5 (7.9%), MSM 5 (7.9%), pregnant women 4 (6.3%), patients living with HIV 4 (6.3%), people with infertility 3 (4.7%), adolescents 1 (1.5%), market vendors 1 (1.5%) and fishermen 1 (1.5%).

#### Serological methods

Most of the 33 serological studies were cross-sectional and comparisons of different ELISA assays in different geographical regions, likely since previous research had found widely varying performances of ELISAs between HIC and certain African populations.^20 21^ Two studies (6.1%) confirmed that ELISA testing had comparable performance to the more expensive Western Blot and therefore is suitable for clinical and epidemiological contexts.^20 21^ 12 studies (36.3%) compared the performance of different ELISA assays, mainly Kalon (Kalon Biological, Guildford, UK) and Herpeselect (Focus Diagnostics, Cypress, California, USA).^22–33^ Most of these studies found the Kalon assay to have the best performance (specificity and sensitivity), and only two found an alternative assay to perform better. However, most studies had to increase the index cut-off value for a positive test to optimise the performance parameters. Three studies (9.1%) evaluated the effect of HIV seropositivity on ELISA performance; two found no effect and one found the specificity of Hereselect to be 30% lower in HIV seropositive people.^22 26 29^ Two studies (6.1%) in East Asia and Western Asia regions found variation in the best-performing assay in high-risk and low-risk groups.^27 28^ Four studies (12.1%) evaluated the rapid point-of-care tests, Biokit (‘Biokit’, Lexington, Massachusetts, USA) and Herpeselect Express Rapid (Focus Technologies, Cypress, California, USA).^22 27 30 34^ All assays produced poor specificities, and their only value was in combination with a standard ELISA assay. Three studies (9.1%) agreed that HSV-2 IgM testing has little value on its own.^35–37^ Other less frequently assessed serological studies focused on umbilical cord IgG, chemiluminescent immunoassay assay, Bioplex 2200, Euoline Western Blot and protein microarray.

#### Molecular biology/DNA detection

16 studies evaluated molecular biology/DNA detection methods. All the DNA detection methods studied performed well and were deemed the preferred direct method of detecting HSV-2 in genital lesions. Most were commercial assays and two were in-house assays.^38 39^ The four studies on multiplex PCR (testing>1 sexually transmitted infection (STI) simultaneously) found it was a sufficiently accurate and reproducible method which is rapid and has potential to work in low resource settings.^40–43^ Two of these used real-time PCR and three used conventional PCR. All the studies on nested PCR (more accurate modification of conventional PCR) found the technique to be more accurate than alternative direct methods.^44–46^ Studies focusing on real-time PCR, which is generally an even more accurate and rapid type of PCR, also found its performance to be superior to other direct methods.^26 47^ Alternative DNA detection methods studied were automated PCR, loop-mediated isothermal amplification (LAMP) and electrochemical DNA biosensors. One study was identified for each, and they were all found to be accurate, rapid and cost-effective means of detecting and typing HSV- 2 DNA especially in low-resource settings.^39 48 49^

#### Syndromic diagnosis

Of the 12 studies that examined the accuracy of syndromic diagnosis of HSV-2 compared with biomedical testing, 7 (58.3%) found this method to lack sufficient accuracy.^50–56^ In high-risk populations such as clinic attendees and MSM, there was poor specificity for syndromic diagnosis, leading to overdiagnosis.^51 55^ In general populations, syndromic diagnosis was found to have very low sensitivity leading to underdiagnosis.^53^ Reported sensitivities ranged from 14.0% in South African youths and 83.0% in clinic attendees in East Asia.^56 57^ Two studies (16.6%) concluded that although syndromic diagnosis was less accurate than formal testing, it was sufficient for a resource-limited clinic setting provided the algorithm was regularly updated according to local STI trends.^57^

#### Sampling techniques

The six studies which examined sampling techniques covered endocervicovaginal swabbing, cervicovaginal lavage, cervicovaginal self-collection and dried blood spots (DBS). A 2003 study comparing the accuracy of cervicovaginal lavage to endocervicovaginal swabbing found cervicovaginal lavage to be more accurate and suitable for detecting HSV-2 in the female genital tract.^58^ Another study assessed the validity of cervicovaginal lavage enriched with cervical swabbing as a sample collection method and found it to be a reliable and accurate technique suitable for future HSV-2 shedding studies.^59^ One study comparing clinician-collected and self-collected cervicovaginal sampling reported comparable accuracy between both techniques and recommended self-collection as a valuable tool in resource-limited settings.^60^ Two studies assessed the performance of DBS instead of serum samples for ELISA assays. The first assessed Kalon (Kalon Biological, Guildford, UK) and found poor accuracy; the second assessed Kalon and Herepselect (Focus diagnostics, Cypress, California, USA) and found poor accuracy for Kalon, but sufficient accuracy for Herpeselect (sensitivity=98.8%, specificity=98.9%).^61 62^

Table 3 demonstrates the extent of the HSV-2 diagnostics research progress according to the associated research priorities set in the 2001 WHO HSV-2 workshop.

**Table 3:** HSV-2 diagnostics research progress according to the associated research priorities set in the 2001 WHO HSV-2 workshop

WHO 2001 research priority: HSV-2 diagnostics	Has the priority been addressed?			Explanation
	Yes	Partially	NO	
Optimal sampling techniques for direct diagnostic methods		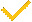		Self-collection validated in two studies, more studies required for enhanced repeatability. Cervicovaginal lavage superior to endocervicovaginal swabbing, one study. Cervicovaginal lavage enhanced with swabbing an accurate method, one study.
Quantification techniques for HSV-2 DNA detection	 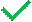	 		Several techniques studies Repeatability between studies DNA detection methods have become the preferred direct technique over the 20-year time period.
Development of a rapid diagnostic test for genital herpes diagnosis	 	  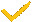  	 	Insufficient accuracy of all assays Only two assays evaluated (Biokit and Herpeselect Rapid) No development of new assay
Performance of existing serological tests in different African sera	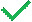 	 		ELlSA assays tested in several African regions/populations. Best performing assay determined Effect of HIV infection on assay performance evaluated. Optimal assay cut-off index values determined

HSV-2, herpes simplex virus type 2.

## Discussion

### Addressed WHO HSV-2 research priorities

Well-defined priorities can avoid waste and bias in the commissioning and conducting of research. Evaluation of research progress against past priority setting is essential to ensure research continues to meet identified needs.^63^ Our comprehensive content analysis aimed to describe the progress made in the prioritised areas of HSV-2 epidemiology and HSV-2 diagnosis from 2000 to 2020.

Over the 20 years period, the published research has undoubtedly reflected the WHO research priorities set in 2001.^64^ Nearly all the research priorities for HSV-2 epidemiology were addressed. This review found that the most frequently studied area was HSV-2 prevalence among the general population. The HSV-2 prevalence appeared to be higher among the Sub-Saharan African population, followed by South America and South and East Asia, respectively. A similar finding was produced by previous global estimates of HSV-2.^1 65 66^ Within sub-Saharan Africa, the highest HSV-2 estimates were reported in the South and West Africa regions compared with the East Africa region, although most of the identified studies (n=14) were based in the latter region. One likely explanation for the lower number of studies from West Africa is our exclusion of non-English language records, given that the official language of many West African countries is French.^67^ Further, population prevalence comparisons should be interpreted with caution given that the overall HSV-2 prevalence is taken from independent studies which are different in terms of the population studied, HSV-2 serological test method and age distribution.

Higher HSV-2 prevalence was found in women compared with men. This gender discrepancy appears constant over different geographical areas and could be due to a higher biological susceptibility to HSV-2 infection in women than men.^68^ Also, as would be expected for a virus that causes lifelong infection, most studies found that HSV-2 prevalence is strongly associated with age.^69^

However, the review’s finding also draws attention to the limited research that explores the reasons for the high HSV-2 prevalence in young adults (mean age=25 years), particularly women from sub-Saharan Africa. The finding also highlights the limited research that examines the contribution of HSV-2 to GUD. Given that HSV-2 infection can lead to recurrent GUD, more studies are required.

We found no HSV-2 studies based in the Middle East, North Africa and Eastern Europe during the studied period; however, because we excluded non-English language records and could not use separate search terms for each developing country, we likely have missed several relevant studies specific to those regions.

All the research priorities for HSV-2 diagnostics were addressed, at least in part. Substantial progress was made in DNA detection methods and exploring the performance of existing serological assays in different African sera. More accurate and rapid DNA detection techniques continue to be researched, and many African countries have ascertained the most accurate ELISA for their populations. However, establishing the optimal genital sampling technique was a research priority that was only partially addressed. The six studies we found showed little repeatability and no definite conclusion. Furthermore, although four studies evaluated the performance of point-of-care assays, they reported poor accuracy.

### Unexamined WHO research priority

Antiviral resistance studies were limited in LMICs (only one study was identified). This may be because multiple LMICs, especially in sub-Saharan Africa, were yet to incorporate aciclovir into their GUD syndromic management algorithm.^70^

### Limitations

The researchers employed a descriptive content analysis approach to measure the progress made in the indicated key areas over 20 years. This enabled them to use classification and tabulation to draw reasonable and reliable conclusions from data records and track progress. However, there are limitations to this method that raise the risk of bias. First, using meta-regressions would be best suited to draw conclusions about differences in effect sizes for various regions and factors such as sex and population type. Second, at the data level, most of the included studies were cross-sectional studies that applied non-probability sampling methods or were conducted in a clinical or hospital setting. Their findings, may, therefore not be representative or generalisable. Finally, our focus on the headline objectives, likely caused us to miss research on more nuanced subthemes, for example, our interest in HSV-2 as a cause of GUD prevented a focus on the interesting question of HSV-2 vs HSV-1 as a cause of GUD.

The search strategy also had its limitations. Due to the time and resource constraints, separate search terms for each developing country could not be used, although the search was conducted with the support of an experienced librarian. Also, EMBASE, a commonly used database was not included in the search strategy because the host institutions did not have access to it. Finally, only English language studies were included. Together, these limitations meant that relevant studies written in English and any other language likely have been excluded. This may particularly affect research published in French since it is the national language of many West and Central African countries.

By contrast, several steps were taken to mitigate as many sources of bias as possible, including independent screening and searching of reference lists. The detailed steps are presented in a companion paper.^16^

Also, a final important limitation is our inability to judge whether or not the WHO HSV-2 priority setting meeting itself directly affected the progress made regarding HSV-2 research in LMICs since only a limited number of articles included in this review cited the original workshop report. This does not preclude the workshop from influencing the commissioning of research by donors or researchers at the funding application stage.

### Areas for consideration for further research

#### HSV-2 epidemiology

While most HSV-2 epidemiology research priorities identified in the workshop were addressed, some gaps remain as follows:

- Some geographical areas have been neglected (eg, North Africa, Middle East, Eastern Europe and Western Asia).
- Causes of the high HSV-2 incidence among younger persons and some at-risk groups (eg,) especially in the African region.
- Understanding the contribution of HSV-2 to GUD in LMICS.

#### HSV-2 diagnostics

All the research priorities for HSV-2 diagnostics in LMICs identified in the workshop were addressed. However, the research priority that emphasises the need to establish the optimal genital sampling techniques for direct diagnostic methods was only partly addressed since only six studies were published over the 20 years period, showing little repeatability and no definite conclusion. Furthermore, although four studies evaluated the performance of two different point-of-care assays for HSV-2 they reported poor accuracy.

## Conclusion

Overall, most HSV-2 research priorities identified by 2001 WHO workshop on HSV-2 epidemiology and HSV-2 diagnostics have been addressed, with the exception of a few priorities relating to point-of-care testing, HSV-2 epidemiology in neglected geographical settings such as North Africa, the Middle East and various populations subgroups.

10.1136/bmjgh-2023-012717.supp4 Supplementary data

## Data Availability

All data relevant to the study are included in the article or uploaded as online supplemental information.
