# Supplementary material for: Twenty years of herpes simplex virus type 2 (HSV-2) research in low-income and middle-income countries: systematic evaluation of progress made in addressing WHO prioritiesfor research in HSV-2 epidemiology and diagnostics
Source: BMJ Glob Health. 2024 Jul 4;9(7):e012717. doi: 10.1136/bmjgh-2023-012717 (PMC11227754; doi:10.1136/bmjgh-2023-012717)
Supplement: Supplementary data [file bmjgh-2023-012717supp001.pdf]

Appendix 1 – Search strategy **(MEDLINE, CINAHL, Global Health, Cochrane)**

Objective 1- HSV-2 Epidemiology

**Database: MEDLINE**		
**Date searched: 01/08/2020**		
**Search number**	**Search terms**	**Number of results**
S10	S3 AND S6 AND S9	467
S9	S7 OR S8	289,280
S8	(MH "Developing Countries")	74,612
S7	“developing countr*” OR “low income countr*” OR LIC OR LMIC OR “low to middle income countr*” OR “low-to-middle income countr*” OR “low-to-middle income” OR “low to middle income” OR “low income” OR “middle income” OR “least developed countr*” OR “less developed countr*” OR “under developed countr*” OR “under developed nation*” OR “poor countr*” OR “third world countr*” OR “third world nation*” OR “least developed nation*” OR “less developed nation*” OR “global south” OR “sub-Saharan Africa” OR “Asia” OR “South America”	289,280
S6	S4 OR S5	4,955,039
S5	(MH "Epidemiology+") OR (MH "Seroepidemiologic Studies+") OR (MH "Prevalence") OR (MH "Cross-Sectional Studies") OR (MH "Cohort Studies+")	2,481,588
S4	“epidemiology” OR “morbidity” OR “prevalence” OR “incidence” OR “mortality” OR “seroepidemiologic*” OR “seroprevalence”	3,662,685
S3	S1 OR S2	81,850
S2	(MH "Herpes Genitalis") OR (MH "Herpesvirus 2, Human") OR (MH "Herpes Simplex+") OR (MH "Herpes Labialis")	25,607
S1	“HSV-2” OR “genital herpes” OR “herpes” OR “herpes genitalis” OR “herpes simplex 2” OR “human simplex virus 2” OR “herpes virus 2”	79,564

**Database: CINAHL**		
**Date searched: 01/08/2020**		
**Search number**	**Search number**	**Search number**
S10	S3 AND S6 AND S9	162
S9	S7 OR S8	82,345
S8	(MH "Developing Countries")	19,539
S7	“developing countr*” OR “low income countr*” OR LIC OR LMIC OR “low to middle income countr*” OR “low-	82,345

	to-middle income countr*" OR "low-to-middle income" OR "low to middle income" OR "low income" OR "middle income" OR "least developed countr*" OR "less developed countr*" OR "under developed countr*" OR "under developed nation*" OR "poor countr*" OR "third world countr*" OR "third world nation*" OR "least developed nation*" OR "less developed nation*" OR "global south" OR "sub-Saharan Africa" OR "Asia" OR "South America"	
S6	S4 OR S5	1,400,852
S5	(MH "Epidemiology+") OR (MH "Seroprevalence Studies") OR (MH "Prevalence") OR (MH "Cross Sectional Studies")	883,168
S4	"epidemiology" OR "morbidity" OR "prevalence" OR "incidence" OR "mortality" OR "seroepidemiologic*" OR "seroprevalence"	935,382
S3	S1 OR S2	15,035)
S2	(MH "Herpesviruses+") OR (MH "Herpes Genitalis")	7,177
S1	"HSV-2" OR "genital herpes" OR "herpes" OR "herpes genitalis" OR "herpes simplex 2" OR "human simplex virus 2" OR "herpes virus 2"	11,345

**Database: Global Health**		
**Date searched: 01/08/2020**		
**Search number**	**Search number**	**Search number**
S10	S3 AND S6 AND S9	1233
S9	S7 OR S8	855,928
S8	DE "Developing Countries" OR DE "Least Developed Countries"	843,726
S7	"developing countr*" OR "low income countr*" OR LIC OR LMIC OR "low to middle income countr*" OR "low-to-middle income countr*" OR "low-to-middle income" OR "low to middle income" OR "low income" OR "middle income" OR "least developed countr*" OR "less developed countr*" OR "under developed countr*" OR "under developed nation*" OR "poor countr*" OR "third world countr*" OR "third world nation*" OR "least developed nation*" OR "less developed nation*" OR "global south" OR "sub-Saharan Africa" OR "Asia" OR "South America"	855,682
S6	S4 OR S5	767,495

S5	DE "epidemiology" OR DE "seroprevalence" OR DE "disease prevalence" OR DE "epidemiological surveys"	353,880
S4	“epidemiology” OR “morbidity” OR “prevalence” OR “incidence” OR “mortality” OR “seroepidemiologic*” OR “seroprevalence”	766,154
S3	S1 OR S2	14,030
S2	DE "Human herpesvirus 2" OR DE "herpes simplex viruses" OR	7,710
S1	"HSV-2" OR “genital herpes” OR “herpes” OR “herpes genitalis” OR “herpes simplex 2” OR “human simplex virus 2” OR “herpes virus 2”	13,869

**Database: Cochrane**		
**Date searched: 01/08/2020**		
**Search number**	**Search number**	**Search number**
S13	S3 AND S6 AND S9	11
S9	S7 OR S8	20419
S8	[Developing Countries]	835
S7	“developing countr*” OR “low income countr*” OR LIC OR LMIC OR “low to middle income countr*” OR “low-to-middle income countr*” OR “low-to-middle income” OR “low to middle income” OR “low income” OR “middle income” OR “least developed countr*” OR “less developed countr*” OR “under developed countr*” OR “under developed nation*” OR “poor countr*” OR “third world countr*” OR “third world nation*” OR “least developed nation*” OR “less developed nation*” OR “global south” OR “sub-Saharan Africa” OR “Asia” OR “South America”	20419
S6	S4 OR S5	45773
S5	[Epidemiology] OR [Prevalence] OR [Seroepidemiologic Studies] OR [Cross-Sectional Studies]	8475
S4	“epidemiology” OR “morbidity” OR “prevalence” OR “incidence” OR “mortality” OR “seroepidemiologic*” OR “seroprevalence”	37698
S3	S1 OR S2	1705
S2	[Herpes Simplex] OR [Herpesvirus 2, Human] OR [Herpes Genitalis]	858

S1	"HSV-2"OR "genital herpes" OR "herpes" OR "herpes genitalis" OR "herpes simplex 2" OR "human simplex virus 2" OR "herpes virus 2"	1457

Objective 2- HSV-2 Diagnostic

**Database: MEDLINE**		
**Date searched: 01/08/2020**		
**Search number**	**Search terms**	**Number of results**
S6	Limiters- full text only	122
S5	Limiters- English only, post year 2000	402
S4	S1 AND S2 AND S3	639
S3	(MH "diagnosis") OR (MH "polymerase chain reaction") OR (MH "cell culture techniques") OR (MH "serologic tests*") OR (MH "serology") OR (MH "immunoassay") OR diagnos* OR "diagnostic test*" OR test* OR "rapid diagnostic test*" OR "rapid test*" OR "point of care test*" OR NAAT OR "Nucleic Acid Amplification Test" OR PCR OR "Polymerase chain reaction" OR immunoassay OR culture OR serolog* OR antibod*	10,037,490
S2	(MH "developing countries") OR "developing countr*" OR LMIC OR "low-to-middle income" OR "low to middle income" OR "low income" OR "middle income"OR "lEast developed countr*" OR "less developed countr*" OR "under developed countr*" OR "under developed nation*" OR "poor countr*" OR "third world countr*" OR "third world nation*" OR "lEast developed nation*" OR "less developed nation*" OR "global South" OR "sub-Saharan Africa" OR Asia OR "South America"	288,482
S1	(MH "Herpes Simplex+") OR (MH "Herpes Labialis") OR (MH "Herpes Genitalis") OR (MH "Herpesvirus 2, Human") OR "herpes virus 2" OR "human simplex virus 2" OR "herpes simplex 2" OR "herpes genitalis" OR herpes OR "genital herpes" OR HSV-2	81,907

Database: CINAHL		
**Date searched: 01/08/2020**		
**Search number**	**Search terms**	**Number of results**
S6	Limiters- full text only	33
S5	Limiters- English only, post year 2000	116
S4	S1 AND S2 AND S3	125
S3	(MH “diagnosis”) OR (MH “polymerase chain reaction”) OR (MH “laboratory diagnosis”) OR diagnos* OR “diagnostic test*” OR test* OR “rapid diagnostic test*” OR “rapid test*” OR “point of care test*” OR NAAT OR “Nucleic Acid Amplification Test” OR PCR OR “Polymerase chain reaction” OR immunoassay OR culture OR serolog* OR antibod*	2,179,651
S2	(MH “developing countries”) OR (MH “low and middle income countries”) OR “developing countr*” OR LMIC OR “low-to-middle income” OR “low to middle income” OR “low income” OR “middle income”OR “lEast developed countr*” OR “less developed countr*” OR “under developed countr*” OR “under developed nation*” OR “poor countr*” OR “third world countr*” OR “third world nation*” OR “lEast developed nation*” OR “less developed nation*” OR “global South” OR “sub-Saharan Africa” OR Asia OR “South America”	82,099
S1	(MH "herpesviruses") OR (MH "Herpes Genitalis") OR (MH “herpes simplex”) OR “herpes virus 2” OR “human simplex virus 2” OR “herpes simplex 2” OR “herpes genitalis” OR herpes OR “genital herpes” OR HSV-2	12,567

Database: Global Health		
**Date searched: 01/08/2020**		
**Search number**	**Search terms**	**Number of results**
S6	Limiters- full text only	
S5	Limiters- English only, post year 2000	2,047
S4	S1 AND S2 AND S3	2,350

S3	(DE “diagnosis”) OR (DE “laboratory diagnosis”) OR (DE “immunodiagnosis”) OR diagnos* OR “diagnostic test*” OR test* OR “rapid diagnostic test*” OR “rapid test*” OR “point of care test*” OR NAAT OR “Nucleic Acid Amplification Test” OR PCR OR “Polymerase chain reaction” OR immunoassay OR culture OR serolog* OR antibod*	1,100,893
S2	(DE “developing countries”) OR (DE “lEast developed countries”) OR (DE “SADC Countries”) OR “developing countr*” OR LMIC OR “low-to-middle income” OR “low to middle income” OR “low income” OR “middle income”OR “lEast developed countr*” OR “less developed countr*” OR “under developed countr*” OR “under developed nation*” OR “poor countr*” OR “third world countr*” OR “third world nation*” OR “lEast developed nation*” OR “less developed nation*” OR “global South” OR “sub-Saharan Africa” OR Asia OR “South America”	969,847
S1	(DE "human herpesvirus 2") OR (DE “Herpes Simplex Viruses”) OR “herpes virus 2” OR “human simplex virus 2” OR “herpes simplex 2” OR “herpes genitalis” OR herpes OR “genital herpes” OR HSV-2	13,918

**Database: Cochrane Library**		
**Date searched: 01/08/2020**		
**Search number**	**Search terms**	**Number of results**
S6	Limiters- full text only	
S5	Limiters- English only, post year 2000	7
S4	S1 AND S2 AND S3	7
S3	(MH “diagnosis”) OR diagnos* OR “diagnostic test*” OR test* OR “rapid diagnostic test*” OR “rapid test*” OR “point of care test*” OR NAAT OR “Nucleic Acid Amplification Test” OR PCR OR “Polymerase chain reaction” OR immunoassay OR culture OR serolog* OR antibod*	437,893

S2	(MH “developing countries”) OR “developing countr*” OR LMIC OR “low-to-middle income” OR “low to middle income” OR “low income” OR “middle income”OR “lEast developed countr*” OR “less developed countr*” OR “under developed countr*” OR “under developed nation*” OR “poor countr*” OR “third world countr*” OR “third world nation*” OR “lEast developed nation*” OR “less developed nation*” OR “global South” OR “sub-Saharan Africa” OR Asia OR “South America”	6,192
S1	(MH "human herpes virus 2") OR “herpes virus 2” OR “human simplex virus 2” OR “herpes simplex 2” OR “herpes genitalis” OR herpes OR “genital herpes” OR HSV-2	4,288
