# Supplementary material for: Twenty years of herpes simplex virus type 2 (HSV-2) research in low-income and middle-income countries: systematic evaluation of progress made in addressing WHO prioritiesfor research in HSV-2 epidemiology and diagnostics
Source: BMJ Glob Health. 2024 Jul 4;9(7):e012717. doi: 10.1136/bmjgh-2023-012717 (PMC11227754; doi:10.1136/bmjgh-2023-012717)
Supplement: Supplementary data [file bmjgh-2023-012717supp002.pdf]

Appendix 2: All the articles included and characteristics.

HSV 2 Epidemiology

Authors	Year	Title	Geographical area of study population (according to UN classification)	Main findings/Prevalence	Journal
Abraham, C. D , Conde Glez, C. J , Cruz Valdez, A , Sánchez Zamorano, L , Hernández Márquez, C ,Lazcano Ponce, E.	2003	Sexual and demographic risk factors for herpes simplex virus type 2 according to schooling level among Mexican youths	Central America	HSV 2 seropositivity was 5.7% , and the ratio of females to males was 2:1	Sexually Transmitted Diseases
Agabi, Y. A , Banwat, E. B , Mawak, J. D , Lar, P. M , Dashe, N , Dashen, M. M , Adoga, M. P , Agabi, F. Y , Zakari, H.	2010	Seroprevalence of herpes simplex virus type 2 among patients attending the Sexually Transmitted Infections Clinic in Jos, Nigeria	West Africa	HSV 2 seropositivity was 141 (87.0%)	Journal of Infection in Developing Countries
Ahmed, H. J , Mbwana, J , Gunnarsson, E , Ahlman, K , Guerino, C , Svensson, L. A , Mhalu, F , Lagergard, T.	2003	Etiology of genital ulcer disease and association with human immunodeficiency virus infection in two Tanzanian cities	East Africa	In Dar es Salaam, HSV 2 prevalnce 63%, in Mbeya, HSV 2 prevalnce 34%	Sexually Transmitted Diseases
Aho, J , Koushik, A , Coutlée, F , Diakité, S. L , Rashed, S.	2014	Prevalence of HIV, human papillomavirus type 16 and herpes simplex virus type 2 among female sex workers in Guinea and associated factors	West Africa	Prevalences of HSV 2 was 84.1%	International Journal of STD & AIDS
Akinyi, B, Odhiambo, C , Otieno, F , Inzaule, S , Oswago, S , Kerubo, E , Ndivo, R , Zeh, C.	2017	Prevalence, incidence and correlates of HSV 2 infection in an HIV incidence adolescent and adult cohort study in western Kenya	East Africa	HSV 2 prevalence was 26.6% [95% confidence interval (CI): 23.9 29.4]. Overall HSV 2 incidence was 4.0 per 100 person years (/100PY) 95% CI: 2.7	Public Library of Sciences (PLoS)

				6.1(MEN 58 (11.8), Female214 (40.2))	
Anjulo, A. A , Tamrat, Abebe , Feleke, Hailemichael , Adane, Mihret	2016	Seroprevalence and risk factors of herpes simplex virus 2 among pregnant women attending antenatal care at health facilities in Wolaita zone, Ethiopia	East Africa	Seroprevalence of HSV 2 infection was 32.1 %	Virology Journal
Anvikara, A. R , Rao, V. G , Savargaonkara, D. D , Yadav, Rajiva , Bhondeleya, M. K , Balkrishna, Tiwaria , Atul, Karkarea , Lukea, C , Vijay, Gadgea , Mahendra, Ukeya , Purushottam, PatelaRao, G. R. R , Ramani, T. V. , Jyothi, Padmaja	2009	Seroprevalence of sexually transmitted viruses in the tribal population of Central India	South Asia	The prevalence of HSV 2 was 20.8% in STI patients compared to 12.4% in the general population.	International Journal of Infectious Diseases
Béhanzin, L , Diabaté, S , Minani, I , Lowndes, C. M , Boily, M. C , Labbé, A. C , Anagonou, S , Zannou, D. M , Buvé, A , Alary, M.Suresh, Malhotra , Tejinder, Kaur , Ahmed, K , Deese, J , Damme, L. van Crucitti, T.	2012	Decline in HIV prevalence among young men in the general population of Cotonou, Benin, 1998 2008	West Africa	In 1998, prevalence of HSV 2 was 21.1 % and In 2008, 26.4 %	PLoS ONE
Benzaken, A , Sabidó, M , Galban, E , Dutra, D. L. R , Leturiondo, A. L , Mayaud, P.	2012	HIV and sexually transmitted infections at the borderlands: situational analysis of sexual health in the Brazilian Amazon	South America	prevalence of HSV 2 was 72.1% femle, male 51.1%	Sexually Transmitted Infections

Brown, J. M , Wald, A , Hubbard, A , Rungruengthanakit, K , Chipato, T , Rugpao, S , Mmiro, F , Celentano, D. D , Salata, R. S , Morrison, C. S , Richardson, B. A , Padian, N. S.Rahman, M , Nahar, S , Ranst, M. van , Verhaegen, J.	2007	Incident and prevalent herpes simplex virus type 2 infection increases risk of HIV acquisition among women in Uganda and Zimbabwe	Multiple regions	HSV 2 seroprevalence was 52% in Uganda and 53% in Zimbabwe. The Seroincidence during follow up was 9.6 and 8.8/100 person years in Uganda and Zimbabwe, respectively.	AIDS
Buvé, A , Caraël, M , Hayes, R. J , Auvert, B , Ferry, B , Robinson, N. J , Anagonou, S , Kanhonou, L , Laourou, M , Abega, S ,Akam, E , Zekeng, L , Chege, J , Kahindo, M , Rutenberg, N , Kaona, F , Musonda, R. Sukwa, T , Morison, L , Weiss, H. A , Laga, M.	2001	The multicentre study on factors determining the differential spread of HIV in four African cities: summary and conclusions	Multiple regions	Cotonous (29%), Yaounde (50%), Kisumu (67.7 %), Ndola (55.%) HIGHER IN WOMEN	AIDS
Caldeira, T. D. M , Gonçalves, C. V , Oliveira, G. R. de , Fonseca, T. V. da , Gonçalves, R , Amaral, C. T. do , Hora, V. P. da , Martinez, A. M. B. de	2013	Prevalence of herpes simplex virus type 2 and risk factors associated with this infection in women in Southern Brazil	South America	The prevalence of HSV 2 was 15.6%.	Revista do Instituto de Medicina Tropical de São Paulo
Cárcamo, C. P ,Campos, P. E ,García, P. J ,Hughes, J. P ,Garnett, G. P ,Holmes, K. K.	2012	Prevalences of sexually transmitted infections in young adults and female sex workers in Peru: a national population based survey	South America	Overall prevalence of HSV2 was 13·5% in men, 13·6% in women, and 60·6% in FSWs	Lancet Infectious Diseases
Celentano, DavidD , Mayer, KennethH , Pequegnat, Willo , Abdala, Nadia , Green, AnnetteM , Handsfield, H. Hunter Hartwell, TylerD , National Institute of Mental Health Collaborative	2010	Prevalence of Sexually Transmitted Diseases and Risk Behaviors from the NIMH Collaborative HIV/STD Prevention Trial	South America	China (M (6.1 %) F (9.6%)), India (M (8.6%) F(56.8%)), Peru (M (24.4%),F (43.5%)), Russia (M (1.4%) F(4%)), Zimbabwe (M (13.5%) F(41.2%))	International Journal of Sexual Health

Charpentier, C , Koyalta, D ,Ndinaromtan, M , Tchobkréo, B , Jenabian, M. A , Day, N , Si Mohamed, A , Weiss, H , Bélec, L.	2011	Distribution of HIV 1 and HSV 2 epidemics in Chad revealing HSV 2 hot spot in regions of high risk HIV spread	Central Africa	HSV 2 seroprevalences was 15.7% (CI95 = 2.9% 24.6%)	Journal of Infection in Developing Countries
Chawla, R , Bhalla, P , Bhalla, K , Singh, M. M ,Garg, S.	2008	Community based study on seroprevalence of herpes simplex virus type 2 infection in New Delhi	South Asia	The prevalence of HSV 2 was found to be 7 and 8.6% in men and women, respectively.	Indian Journal of Medical Microbiology
Chen, XiangSheng , Yin, YuePing , Chen, LeiPing , Yu, YanHua , Wei, WanHui , Nguyen Thi Thanh, Thuy , Smith, J. S.	2007	Herpes simplex virus 2 infection in women attending an antenatal clinic in Fuzhou, China	East Asia	HSV 2 seroprevalence was 10.8% (95% CI: 8.3% to 13.8%)	Sexually Transmitted Infections
Chen, XiangSheng, Yin, YuePing , Gong, XiangDong , Liang, GuoJun , Zhang, WenYing , Poumerol, G , Shi, MeiQin , Wu, ShouQiang , Zhang, GuoCheng	2006	Prevalence of sexually transmitted infections among long distance truck drivers in Tongling, China	East Asia	HSV 2 in 4.4%	International Journal of STD & AIDS
Chow, E. P. F ,Tucker, J. D , Wong, F. Y , Nehl, E. J , Wang, YanJie , Zhuang, Xun , Zhang, Lei	2014	Disparities and risks of sexually transmissible infections among men who have sex with men in China: a meta analysis and data synthesis	East Asia	The prevalence of HSV 2 was 10.6% (CL 6.2–17.6%)	PLoS ONE
Clark, J. L , Konda, K. A , Munayco, C. V , Pún, M , Lescano, A. G , Leon, S. R , Pajuelo, J , Suarez Ognio, L , Klausner, J. D , Coates, T. J ,Cáceres, C. F.	2008	Prevalence of HIV, herpes simplex virus 2, and syphilis in male sex partners of pregnant women in Peru	South America	Prevalence HSV 2 was 16.0% (95%CI 14.3%–17.8%)	BMC Public Health

Conde Glez, C , Lazcano Ponce, E , Rojas, R , DeAntonio, R , Romano Mazzotti, L , Cervantes, Y , Ortega Barria, E.	2013	Seroprevalences of varicella zoster virus, herpes simplex virus and cytomegalovirus in a cross sectional study in Mexico	Central America	HSV 2 seroprevalences was 9.9% (95% CI: 7.9−12.0). Female higher in men	Vaccine
Couture, M. C , Soto, J. C , Akom, E , Labbé, A. C , Joseph, G , Zunzunegui, M. V.	2008	Clients of female sex workers in Gonaives and St Marc, Haiti characteristics, sexually transmitted infection prevalence and risk factors	Caribbean	The prevalence of HSV 2 was 22%, P value = 0.001	Sexually Transmitted Diseases
Cowan, F. M , French, R. S , Mayaud, P , Gopal, R , Robinson, N. J , Oliveira, S. A. de , Faillace, T , Uusküla, A , Nygård Kibur, M , Ramalingam, S , Sridharan, G ,El Aouad, R , Alami, K , Rbai, M , Sunil Chandra, N. P , Brown, D. W.	2003	Seroepidemiological study of herpes simplex virus types 1 and 2 in Brazil, Estonia, India, Morocco, and Sri Lanka	Multiple regions	Barazil, ANC attenders (29.3%), Estonia (23.8%), India (7.9%), M arocco (12.9%%), Sri Lanka (8.3%) higher in women	Sexually Transmitted Infections
Daniels, B , Wand, H , Ramjee, G.	2016	Prevalence of Herpes Simplex Virus 2 (HSV 2) infection and associated risk factors in a cohort of HIV negative women in Durban, South Africa	Southren Africa	The HSV 2 prevalence at base line in this cohort of women was 65%. The HSV 2 incidence during study follow up was 22.3 per 100 PY (95% CI 20.4–24.3)	BMC Research Notes
Dave, S. S , Copas, A , Richens, J , White, R. G ,. Kosambiya, J. K , Desai, V. K , Stephenson, J. M.	2012	HIV and STI prevalence and determinants among male migrant workers in India	South Asia	Herpes simplex virus 2 w were 3.3 (2.0–5.4), 3.5 (2.1–5.9) , 3.4 (2.4–4.9) among different	PLoS ONE
Lai,W., Chen,C.Y., Morse,S. A., Fehler, H.G. , Liu, H., Ballard, R.C.	2003	Increasing relative prevalence of HSV 2 infection among men with genital ulcers from a mining community in South Africa	Southren Africa	M PCR detectedT pallidum, HSV, andH ducreyiin 10.3%, 17.2%, and 69.4% of 232 GUDpatients during 1993–4 and in 12.4%,	Sexually Transmitted Infections

				36.0%, and 50.5% of 186 GUD patients in 1998. The pro portion of patients with more than one agent increased significantly from 7.3% (17/232) in 1993–4to 16.7% (31/186) in 1998 (p <0.01). HSV 2 was detected in a higher proportion of ulcer specimensfrom HIV infected patients than in specimens from HIV uninfected patients during both time periods(1993–4: 26.2%v6.7%, p <0.001; 1998: 42.1%v29.6%, p >0.09).	
Davies, Stephen C, Taylor, Janette A , Sedyaningsih Mamahit, Endang R , Gunawan, Suriadi , Cunningham, Anthony L , Mindel, Adrian	2007	Prevalence and risk factors for herpes simplex virus type 2 antibodies among low and high risk populations in Indonesia	East Asia	HSV 2 antibodies were detected in 153 of 176 FSWs (86.9%; 95% confidence interval [CI], 81.0–91.5); among nonsex worker females, HSV 2antibodies were detected in 78 of 418 (18.7%; 95% CI, 15.0–22.7)	Sexually Transmitted Diseases
Debrah, Oksana , Agyemang Yeboah, Francis , Asmah, Richard Harry , Timmy Donkoh, Emmanuel ,Seini, Mohammed Mustapha , Fondjo, Linda Ahenkorah , Sight, Nilok , Owusu Dabo, Ellis	2018	SERO prevalence of herpes simplex virus type 1 and type 2 among women attending routine Cervicare clinics in Ghana	West Africa	The HSV 2 seroprevalence estimates was 78.4% (95% CI:74.5–81.8%)	BMC Infection disease

Desta, Kassa , Gebremedhin, Gebremichael , Tesfaye, Tilahun , Abenezer, Ayalkebet , Yemane, Abrha , Getnet, Mesfin , Yohannes, Belay , Minilik, Demissie , Atsbeha, Gebrexiabher , Yibeltal, Assefa	2018	Prevalence of sexually transmitted infections (HIV, hepatitis B virus, herpes simplex virus type 2, and syphilis) in pregnant women in Ethiopia: trends over 10 years (2005 2014)	East Africa	The prevalence of HSV 2 range between (47.5% to 28.5%)	International Journal of Infectious Diseases
Dipankar, Biswas , Biswajyoti, Borkakoty , Jagadish, Mahanta , Kamini, Walia , Lahari, Saikia , Akoijam, B. S , Lobsang, Jampa , Alia, Kharkonggor , Eric, Zomawia	2011	Seroprevalence and risk factors of herpes simplex virus type 2 infection among pregnant women in Northeast India	South Asia	seroprevalence of HSV 2 infection was 8.7% (95% CI 7.3 10.0)	BMC Infection disease
Domercant, J. W, Louis, F. J , Hulland, E , Griswold, M , Andre Alboth, J , Ye, Tun , Marston, B. J.	2017	Seroprevalence of Herpes Simplex Virus type 2 (HSV 2) among pregnant women who participated in a national HIV surveillance activity in Haiti	Caribbean	The prevalence of HSV 2 was 31.4% (95% CI: 27.7–35.4)	BMC Infection disease
Drisu, U. I, Oronsaye, F. E , Adejumo, B. I. G ,Uchuno, G. A , Emmanuel, A. M , Dimkpa, U , Omosor, K. I , Adebowale, M. O , Abdulrahman, O. N , Ukatu, E. N.	2018	Seroprevalence, type specific of herpes simplex virus and associated risk factors among women of child bearing age in Kogi state, Nigeria	West Africa	The prevalence of HSV was 76.7%.	Health
Duan, S , Ding, Y , Wu, Z , Rou, K , Yang, Y , Wang, J , Gao, M , Ye, R , Xiang, L , He, N.	2016	The prevalence of HSV 2 infection in HIV 1 discordant couples	East Asia	The prevalence of HSV was 42·4%	Epidemiology and Infection
Eis Hübinger, A. M , Nyankiye, E , Bitoungui, D. M , Ndjomou, J.	2002	Prevalence of herpes simplex virus type 2 antibody in Cameroon	Central Africa	the HSV 2 seroprevalence was 70.0%.	Sexually Transmitted Diseases

Feng, Y , Wu, ZunYou , Detels, R , Qin, GuangMing , Liu, Li , Wang, XiaoDong , Wang, Jun , Zhang, LingLin	2010	HIV/STD prevalence among men who have sex with men in Chengdu, China and associated risk factors for HIV infection	East Asia	HSV 2 prevalence was , 24.7%	JAIDS, Journal of Acquired Immune Deficiency Syndromes
Finger Jardim, F , Avila, E. C , Hora, V. P. da , Gonçalves, C. V , Martinez, A. M. B. de , Soares, M. A.	2017	Prevalence of herpes simplex virus types 1 and 2 at maternal and fetal sides of the placenta in asymptomatic pregnant women	South America	The prevalence of HSV -2 was 12.6% (maternal side) and 8.3% (fetal side).	American Journal of Reproductive Immunology
Francis, Suzanna C , Mthiyane, T. Nondumiso , Baisley, Kathy , McHunu, S. Lerato , Ferguson, Jane B , Smit, Theresa , Crucitti, Tania , Gareta, Dickman , Dlamini, Siphephelo , Mutevedzi, Tinofa , Seeley, Janet , Pillay, Deenan , McGrath, Nuala , Shahmanesh, Maryam	2018	Prevalence of sexually transmitted infections among young people in South Africa: A nested survey in a health and demographic surveillance site	Southren Africa	the prevalence of HSV 2 among men and women, respectively, was 16.8% and 28.7%	PLoS ONE
Gabster, A , Pascale, J. M , Cislaghi, B , Francis, S. C , Weiss, H. A , Martinez, A , Ortiz, A , Herrera, M , Herrera, G , Gantes, C. , Quiel, Y , Ríos, A , Campbell, E , Mayaud, P.	2019	High prevalence of sexually transmitted infections, and high risk sexual behaviors among indigenous adolescents of the Comarca Ngäbe Buglé, Panama	Central America	in HSV 2 seroprevalence (females, 16.1%; males, 16.1%;P= 0.99).	Sexually Transmitted Diseases
Gibney, L , Saquib, N ,Macaluso, M , Hasan, K. N , Aziz, M. M , Khan, A. Y. M. H ,Choudhury, P.	2002	STD in Bangladesh's trucking industry: prevalence and risk factors	South Asia	The prevalence of HSV 2 was (25.8%)	Sexually Transmitted Infections

Gutierrez, J. P , Bertozzi, S. M , Conde Glez, C. J , Sanchez Aleman, M. A.	2006	Risk behaviors of 15 21 year olds in Mexico lead to a high prevalence of sexually transmitted infections: results of a survey in disadvantaged urban areas	Central America	The prevalence of HSV 2 was 12%	BMC Public Health
Gutierrez, J. P , Conde González, C. J , Walker, D. M , Bertozzi, S. M.	2007	Herpes simplex virus type 2 among Mexican high school adolescents: prevalence and association with community characteristics	Central America	The seropositivity of HSV 2 was 21%	Elsevier
Halton, K , Ratcliffe, A. A , Morison, L , West, B , Shaw, M , Bailey, R , Walraven, G.	2003	Herpes simplex 2 risk among women in a polygynous setting in rural West Africa	West Africa	HSV2 prevalence was 16% amongst never married women and 36% amongst ever married women	AIDS
Hazel, A , Foxman, B , Low, B. S.	2015	Herpes simplex virus type 2 among mobile pastoralists in northwestern Namibia	Southren Africa	The overall prevalence of HSV 2 was 35%	Annals of Human Biology
Hoa Van, Le , Schoenbach, V. J , Herrero, R , Anh Thi Hoang, Pham , Hieu Trong, Nguyen , Thuy Thi, Nguyen , Muñoz, N , Franceschi, S , Vaccarella, S , Parkin, M. D , Snijders, P. J. F , Morrow, R. A. , Smith, J. S.	2009	Herpes simplex virus type 2 seropositivity among ever married women in South and North Vietnam: a population based study	East Asia	HSV 2 seroprevalence was (30.8%, 95% CI: 28.1–33.4)	Sexually Transmitted Diseases
Holtz, T. H , Thienkrua, W , McNicholl, J. M , Wimonsate, W , Chaikummao, S , Chonwattana, W , Wasinrapee, P , Varangrat, A , Mock, P. A , Sirivongrangson, P , Griensven, F. van	2012	Prevalence of Treponema pallidum seropositivity and herpes simplex virus type 2 infection in a cohort of men who have sex with men, Bangkok, Thailand, 2006 2010	East Asia	HSV 2 seropositive rates was 20.7%	International Journal of STD & AIDS

Hu, QingHai , Xu, JunJie , Chu, ZhenXing , Zhang, Jing , Yu, YanQiu , Yu, Huan , Ding, HaiBo , Jiang, YongJun , Geng, WenQing , Wang, Ning , Shang, Hong	2017	Prevalence and determinants of herpes simplex virus type 2 (HSV 2)/syphilis co infection and HSV 2 mono infection among human immunodeficiency virus positive men who have sex with men: a cross sectional study in northeast China	East Asia	The prevalence of HSV 2 was 48.6% (95% CL 44.4–52.8%)	Japanese Journal of Infectious Diseases
Huai, PengCheng , Li, FuRong , Li, Zhen , Sun, LeLe , Fu, Xi'an , Pan, Qing , Yu, GongQi , Chai, ZeMin , Chu, TongSheng , Mi, ZiHao , Bao, FangFang , Wang, HongLei , Zhou, BingNi , Wang, Chuan , Sun, YongHu , Niu, GuiYe , Zhang, Yuan , Fu, FangHui , Lang, XiaoQiao , Wang, XiaoLing , Zhao, Hui , Liu, DaiNa , Liu, Hong , Liu, DianChang , Liu, Jian , Xu, AiQiang	2019	Seroprevalence and associated factors of HSV 2 infection among general population in Shandong Province, China	East Asia	The seroprevalence of HSV 2 infection was 4.2% (95% confidence interval [CI], 3.2–5.3) in females, and in males 2.7% (95% CI, 1.1–4.2)	BMC Infection disease
Ibrahim, A , Adamu, I. A , Haruna, A.	2015	Seroepidemiology of Herpes Simplex Virus Type 2 (HSV 2) among incarcerated population of Potiskum medium security prison Potiskum Yobe State: study of prevalence and associated risk factors	West Africa	The seropositive for HSV 2 was 14.5%	International Journal of Current Microbiology and Applied Sciences
Janbakhash, A , Mansouri, F , Vaziri, S , Sayad, B , Afsharian, M , Abedanpor, A.	2012	Seroepidemiology of herpes simplex virus type 2 (HSV2) in HIV infected patients in Kermanshah Iran	South Asia	Out of 170 cases, 11 were seropositive for HSV2 (6.5%) in case group and 2 of 165 (1.21%) in control group (p=0.015).	Caspian Journal of Internal Medicine (CJIM)

Kapiga, S. H , Sam, N. E , Shao, J. F , Masenga, E. J , Renjifo, B , Kiwelu, I. E , Manongi, R , Fawzi, W. W ,Essex, M.	2003	Herpes simplex virus type 2 infection among bar and hotel workers in Northern Tanzania: prevalence and risk factors	East Africa	The seroprevalence of HSV 2 was 43.5%.	Sexually Transmitted Diseases
Karad, A. B , Khade, S. L.	2013	Seroepidemiological study of herpes simplex virus type 2 infection in HIV positive patients	South Asia	Seroprevalence of HSV 2 was 48.4%	International Journal of Medicine and Public Health
Karim, Q. A , Kharsany, A. B. M , Leask, K , Ntombela, F , Humphries, H , Frohlich, J. A , Samsunder, N , Grobler, A , Dellar, R , Karim, S. S. A.	2014	Prevalence of HIV, HSV 2 and pregnancy among high school students in rural KwaZulu Natal, South Africa: a bio behavioural cross sectional survey	Southren Africa	HSV 2 prevalence was 2.6% (95% CI 1.6 to 3.7) in males and 10.7% (95% CI 8.8 to 12.6) in females (p<0.001).	Sexually Transmitted Infections
Kirakoya Samadoulougou, F , Nagot, N , Defer, M. C ,Yaro, S , Fao, P , Ilboudo, F , Langani, Y , Meda, N , Robert, A.	2011	Epidemiology of herpes simplex virus type 2 infection in rural and urban Burkina Faso	West Africa	The prevalence of HSV 2 among pregnant women was 18.0% (95% (CI) 16.3% 19.7%), versus semiurban areas (21.4%; CI: (17.2% 26.2%) (P = 0.08), whereas the prevalence of HSV 2 was 23.7% (CI: 20.9% 26.6%).	Sexually Transmitted Diseases
Konda, Kelika A , Klausner, Jeffrey D , Lescano, Andres G , Leon, Segundo , Jones, Franca R , Pajuelo, Jose , Caceres, Carlos F , Coates, Thomas J.	2005	The epidemiology of herpes simplex virus type 2 infection in low income urban populations in coastal Peru	South America	In the socially marginalized population, HSV 2 prevalencewas 72.3% . In the general population, HSV 2 preva lence was 20.5% in women and 7.1% in men.	Sexually Transmitted Diseases

Kularatne, R. S , Muller, E. E , Maseko, D. V , Kufa Chakezha, T ,Lewis, D. A.	2018	Trends in the relative prevalence of genital ulcer disease pathogens and association with HIV infection in Johannesburg, South Africa, 2007 2015	Southren Africa	Seroprevalence of HSV 2 was 65.2%	PLoS ONE
Li, JiaMin , Chen, YongRu , Li, XiTao , Xu, WenCan	2011	Screening of Herpes simplex virus 2 infection among pregnant women in southern China	East Asia	The prevalence of HSV 2 was 23.56% (95% [CI] = 21.53–26.00)	Journal of Dermatology
Looker, Katharine J , Magaret, Amalia S , Turner, Katherine M. E , Vickerman, Peter , Gottlieb, Sami L , Newman, Lori M.	2015	Global estimates of prevalent and incident herpes simplex virus type 2 infections in 2012	Multiple regions	The prevalence of HSV 2 world wide was 11.3%	PLoS ONE
Lowe, S , Mudzviti, T , Mandiriri, A , Shamu, T , Mudhokwani, P , Chimbetete, C , Luethy, R , Pascoe, M.	2019	Sexually transmitted infections, the silent partner in HIV infected women in Zimbabwe	Southren Africa	HSV 2 prevalence 52.5%	Southern African Journal of HIV Medicine
Luther, K. M. M , Rodrigue, D. N. R , Annie, N. N. R , Jean, A. A , Michel, W , Elisée, E , Henri, A. Z. P.	2015	Seroprevalence and factors associated with herpes simplex virus type 2 and Chlamydia trachomatis among women attending AIDS care unit at the Bonassama district hospital	Central Africa	.The prevalence of HSV 2 n HIV positive patients was 73.7% versus 81.8% in HIV negative individuals.	British Journal of Medicine and Medical Research
Madebe, R , Kiwelu, I , Ndaro, A , Francis, F , Baraka, V , Theilgaard, Z ,Katzenstein, T.	2020	Herpes Simplex virus type 2 seroprevalence and risk factors among adolescents and youth with HIV 1 in Northern, Tanzania	East Africa	The HSV 2 seroprevalence was 18%	Journal of Infection in Developing Countries

Malary, M , Abedi, G , Hamzehgardeshi, Z , Afshari, M , Moosazadeh, M.	2016	The prevalence of herpes simplex virus type 1 and 2 infection in Iran: a meta analysis	South Asia	The total prevalences of HSV 2 was 6.5% (4.7 8.2)	International Journal of Reproductive BioMedicine
Meque, I , Dubé, K , Feldblum, P. J , Clements, A. C. A , Zango, A , Cumbe, F , Chen, P. L , Ferro, J. J , Wijgert, J. H. van de	2014	Prevalence, incidence and determinants of herpes simplex virus type 2 infection among HIV seronegative women at high risk of HIV infection: a prospective study in Beira, Mozambique	Southren Africa	The HSV 2 prevalence at baseline was 60.6% (95% CI: 55.7% –65.4%).	PLoS ONE
Mmbaga, E. J , Moen, K , Makyao, N , Mpembeni, R , Leshabari, M. T.	2017	HIV and STI s among men who have sex with men in Dodoma municipality, Tanzania: a cross sectional study	East Africa	The seroprevalence of HSV 2 was 38.5%,	Sexually Transmitted Diseases
Mohammed, Qutub , Javed, Akhter	2003	Epidemiology of genital herpes (HSV 2) among brothel based female sex workers in Bangladesh	South Asia	a 33 (7.1%) samples were seropositive for HSV	European Journal of Epidemiology
Msuya, S. E , Mbizvo, E , Akhtar, Hussain , Sam, N. E , Jeansson, S , Stray Pedersen, B.	2003	Seroprevalence and correlates of herpes simplex virus type 2 among urban Tanzanian women	East Africa	The seroprevalence of HSV 2 was 39%	Sexually Transmitted Diseases
Mugo, N , Dadabhai, S. S , Bunnell, R , Williamson, J , Bennett, E , Baya, I , Akinyi, N , Mohamed, I , Kaiser, R.	2011	Prevalence of herpes simplex virus type 2 infection, human immunodeficiency virus/herpes simplex virus type 2 coinfection, and associated risk factors in a national, population based survey in Kenya	East Africa	HSV 2seroprevalence was 35.1%	Sexually Transmitted Diseases

Nakku Joloba, E , Kambugu, F , Wasubire, J , Kimeze, J , Salata, R , Albert, J. M , Rimm, A , Whalen, C.	2014	Sero prevalence of herpes simplex type 2 virus (HSV 2) and HIV infection in Kampala, Uganda	East Africa	The HSV 2 prevalence was 58% (95% CI: 55, 60)	African Health Sciences
Nakubulwa, S , Kaye, D. K , Bwanga, F , Tumwesigye, N. M , Nakku Joloba, E , Mirembe, F. M.	2016	Incidence and risk factors for herpes simplex virus type 2 seroconversion among pregnant women in Uganda: a prospective study	East Africa	Of 191 women, 15 (7.9%) seroconverted during pregnancy.	Journal of Infection in Developing Countries
Navadeh, S , Mirzazadeh, A , Mousavi, L , Haghdoost, A. A , Fahimfar, N , Sedaghat, A.	2012	HIV, HSV2 and syphilis prevalence in female sex workers in Kerman, South East Iran; using respondent driven sampling	South Asia	The prevalence of HSV2 was 18.0%	Iranian Journal of Public Health
Ngo, T. D , Laeyendecker, O , Li, C , Tai, H , Cui, M , Lai, S , Quinn, T. C.	2008	Herpes simplex virus type 2 infection among commercial sex workers in Kunming, Yunnan Province, China	East Asia	the HSV 2 prevalence was 33.0%	International Journal of STD & AIDS
Paz Bailey, G , Morales Miranda, S , Jacobson, J. O , Gupta, S. K , Sabin, K , Mendoza, S , Paredes, M , Alvarez, B ,Monterroso, E.	2009	High rates of STD and sexual risk behaviors among Garifunas in Honduras	Central America	The prevalences of HSV 2 was 51.1% (95% CI: 46.7 to 55.6)	JAIDS, Journal of Acquired Immune Deficiency Syndromes
O'Farrell, N , Nguyen Vu, Thuong , Khuu Van, Nghia , Luong Thu, Tram , Nguyen Thanh, Long	2006	HSV2 antibodies in female sex workers in Vietnam	East Asia	The prevalence of HSV 2 antibodies was 27.7% (95% confidence interval [CI]: 24.8–30.7%).	International Journal of STD & AIDS

Panchanadeswaran, S , Johnson, S. C , Mayer, K. H , Srikrishnan, A. K , Sivaran, S , Zelaya, C. E , Go, V. F , Solomon, S , Bentley, M. E , Celentano, D. D.	2006	Gender differences in the prevalence of sexually transmitted infections and genital symptoms in an urban setting in southern India	South Asia	The most commonly detected STI was HSV2; 13.2%	Sexually Transmitted Infections
Paz Bailey, G , Shah, N , Creswell, J , Guardado, M. E , Nieto, A. I , Estrada, M. C , Cedillos, R , Pascale, J. M , Monterroso, E.	2012	Risk behaviors and STI prevalence among people with HIV in El Salvador	Central America	The prevalence of HSV2 was 84.5 %	Open AIDS Journal
Reward, E. E , Muo, S. O , Orabueze, I. N. A , Ike, A. C.	2019	Seroprevalence of herpes simplex virus types 1 and 2 in Nigeria	West Africa	The seroprevalences for HSV 2 was 63.4% (56.1–70.4%).	Pathogens and global health
Rodrigues, J , Grinsztejn, B , Bastos, F. I , Velasque, L , Luz, P. M , Souza, C. T. V. de , Georg, I , Pilotto, J. H , Veloso, V. G.	2009	Seroprevalence and factors associated with herpes simplex virus type 2 among HIV negative high risk men who have sex with men from Rio de Janeiro, Brazil: a cross sectional study	South America	Seroprevalence of HSV 2 was of 45.7%	BMC Infection disease
Rodríguez, A. C , Castle, P. E , Smith, J. S , Bratti, C , Hildesheim, A , Schiffman, M Viscidi, R , Burk, R. D , Ashley, R. L , Castellsagué, X , Herrero, R.	2003	A population based study of herpes simplex virus 2 seroprevalence in rural Costa Rica	Central America	Overall age adjusted HSV 2 seroprevalence was 38.5% (95% CI, 37.5 to 39.5)	Sexually Transmitted Infections
Sanchez Aleman, M. A , Villar Tapia, Y. G. del , Gutierrez, J. P , Garcia Cisneros, S , Olamendi Portugal, M. L , Herrera Ortiz, A , Velazquez Meza, M , Conde Glez, C. J.	2018	Heterogeneity of herpes simplex virus type 2 seroprevalence from a national probability survey in Mexico, 2012	Central America	National HSV 2 seroprevalence was 9.9%, 2.4% among adolescents and 11.7% among adults with HSV 2 seroprevalence heterogeneous across the country.	Sexually Transmitted Diseases

Saramma, Jacob , Thatchinamoorthy, Gopa , Sivasangeetha, Kanagasaba , Anitha, Durairaj , Kamala, Sushi , Ganesan, Arumugam	2015	Herpes simplex virus 2 infection in HIV seropositive individuals in Tamil Nadu, India	South Asia	The overall prevalence of HSV 2 was 50%	International Journal of Medicine and Public Health
Sgaier, S. K , Mony, P , Jayakumar, S , McLaughlin, C , Arora, P , Kumar, R , Bhatia, P , Jha, P.	2011	Prevalence and correlates of Herpes Simplex Virus 2 and syphilis infections in the general population in India	South Asia	The prevalence of HSV 2 was 10.1%.	Sexually Transmitted Diseases
Smith, Jennifer S , Robinson, N. Jamie	2002	Age specific prevalence of infection with herpes simplex virus types 2 and 1: a global review	Multiple regions	HSV 2 prevalnce was in the late 1970s (16.0%) and early 1990s (20.8%)	The Journal of infectious diseases
Sudfeld, C. R , Hewett, P. C , Abuelezam, N. N , Chalasani, S , Soler Hampejsek, E , Kelly, C. A , Mensch, B. S.	2013	Herpes simplex virus type 2 cross sectional seroprevalence and the estimated rate of neonatal infections among a cohort of rural Malawian female adolescents	Southren Africa	11.2% (95% CI 7.4% to 15.9%) for 17 yr old, 15.7% (95% CI 11.9% to 20.2%) for 18 yr old, 17.8% (95% CI 13.4% to 22.9%) for 19 yr old, 18.6% (95% CI 11.9% to 27.0%) for 20 yr old	Sexually Transmitted Infections
Sutcliffe, S , Taha, T. E , Kumwenda, N. I , Taylor, E , Liomba, G. N.	2002	HIV 1 prevalence and herpes simplex virus 2, hepatitis C virus, and hepatitis B virus infections among male workers at a sugar estate in Malawi	Southren Africa	overall HSV 2 prevalence was 69.3 % where the prevalnce was 88.1 % among HIV positive and 64.3 % among negative subject	JAIDS, Journal of Acquired Immune Deficiency Syndromes
Tayyebi, D , Sharifi, S.	2010	Seroepidemiology of infection with herpes simplex virus types 1 and 2 (HSV1 and HSV2) among asymptomatic university students	South Asia	HSV 2 prevalnce was 23.3%	Iranian Journal of Clinical Infectious Diseases

		attending Islamic Azad University of Kazeroun, southwest of Iran			
Theng, C. T. S , Sen, P. R , Chio, T. W. M , Tan, H. H , Wong, M. L , Chan, R. K. W.	2006	Seroprevalence of herpes simplex virus 1 and 2 in attendees of a sexually transmitted infection clinic in Singapore	East Asia	HSV 2 seroprevalnce (28.5%)	sexuall health
Theng, T. S. C , Sen, P. R , Tan, H. H , Wong, M. L , Chan, K. W. R.	2006	Seroprevalence of HSV 1 and 2 among sex workers attending a sexually transmitted infection clinic in Singapore	East Asia	HSV 2 seroprevalnce (79.0%)	International Journal of STD & AIDS
Torrone, E. A , Morrison, C. S , Chen, PaiLien , Kwok, C , Francis, S. C , Hayes, R. J , Looker, K. J , McCormack, S , McGrath, N , Wijgert, J. H. H. M. van d , Watson Jones, D , Low, N , Gottlieb, S. L.	2018	Prevalence of sexually transmitted infections and bacterial vaginosis among women in sub Saharan Africa: an individual participant data meta analysis of 18 HIV prevention studies	Multiple regions	1 HSV 2 prevalence was high among 15–24 year olds, in the South African clinic/community based populations (summary estimate: 39.3% [95%CI:34.3%,44.6%] ;I2= 88.8%), in Southern/Eastern African clinic/community based populations 46.8%[95%CI:38.2%,55.6%], in the higher risk populations in Eastern Africa 56.3%[95%CI:49.2%,63.1%]. 2 HSV 2 was even higher among women aged 25–49 years, in the South African clinic/community based populations	PLoS Medicine

				77.8%(95%CI:75.6%,79.9%), in Southern/Eastern African clinic/community based populations (70.0%[95%CI:61.8%,77.1%), in the higher risk populations in Eastern Africa (83.3%[95%CI:79.3%,86.6%).	
Vandenhoudt, H. M , Langat, L , Menten, J , Odongo, F , Oswago, S , Luttah, G , Zeh, C , Crucitti, T , Laserson, K , Vulule, J , Buve, A.	2007	HIV and other sexually transmitted infections among female sex workers in Kinshasa, Democratic Republic of Congo, in 2002	East Africa	The HSV 2 seroprevalence was 58.5%.	PLoS Medicine
Wang, HaiBo , Wang, Ning , Chen, R. Y , Sharp, G. B , Ma, YanLin , Wang, GuiXian , Ding, GuoWe , Wu, ZhengLai	2008	Prevalence and predictors of herpes simplex virus type 2 infection among female sex workers in Yunnan Province, China	East Asia	HSV 2 was the most common STI (68%)	International Journal of STD & AIDS
Wang, JunJie , Zhu, ZhiBin , Yang, Xi , Wu, Jing , Wang, HaiBo , Feng, Lin , Ding, GuoWei , Norris, J. L , Wang, Ning	2012	Herpes simplex virus type 2 risks in female sex workers in the China Vietnam border county of Hekou	East Asia	HSV 2 prevalence rates were 58.3%	Biomedical and Environmental Sciences
Yahya Malima, K. I , Evjen Olsen, B , Matee, M. I , Fylkesnes, K , Haarr, L.	2008	HIV 1, HSV 2 and syphilis among pregnant women in a rural area of Tanzania: prevalence and risk factors	East Africa	HSV 2 prevalence 20.7% (95% CI: 18.53–23.00)	BMC Infectious Diseases

Yang, Ying , Yao, JinJian , Gao, MeiYang , Su, HuaLin , Zhang, TieJun , He, Na	2011	Herpes simplex virus type 2 infection among female sex workers in Shanghai, China	East Asia	The overall HSV 2 seroprevalence was 47.3% (375/793)	AIDS Care
Yin, YuePing , Chen, ShaoChun , Wang, HongChun , Wei, WanHui , Wang, QianQiu , Liang, GuoJun , Jiang, Ning , Han, Yan , Chen, XiangSheng , Wang, BaoXi	2012	Prevalence and risk factors of HSV 2 infection and HSV 2/HIV coinfection in men who have sex with men in China: a multisite cross sectional study	East Asia	16.0% (95% confidence interval [CI], 14.2%–18.0%)	Sexually Transmitted Diseases
Vu, Lung , Misra, Kavita	2018	High Burden of HIV, Syphilis and HSV 2 and Factors Associated with HIV Infection Among Female Sex Workers in Tanzania: Implications for Early Treatment of HIV and Pre exposure Prophylaxis (PrEP)	East Africa	The prevalence of HSV 2 was 57.5 %	Journal of Infection in Developing Countries
Looker, Katharine J , Welton, Nicky J , Sabin, Keith M , Dalal, Shona , Vickerman, Peter , Turner, Katherine M. E , Boily, Marie Claude , Gottlieb, Sami L.	2020	Global and regional estimates of the contribution of herpes simplex virus type 2 infection to HIV incidence: a population attributable fraction analysis using published epidemiological data	Multiple regions	The prevalence of HSV 2 was 26·8% [19·7–34·5]	The Lancet Infectious Diseases
Paz Bailey, G , Ramaswamy, M , Hawkes, S. J , Geretti, A. M.	2006	Herpes simplex virus type 2: epidemiology and management options in developing countries	Multiple regions	HSV2 have been observed in sub-Saharan Africa, with age-adjusted prevalences in adults ranging from 30% to 80% in women and 10% to 50% in men. In South America, available data are mainly for women, in whom HSV2 prevalence ranges from 20% to 40%. Prevalence in the general population in Asian	Postgraduate medical journal

				countries shows lower values, from 10% to 30%.6	
Madhivanan, Purnima , Krupp, Karl , Chandrasekaran, Varalakshmi , Karat, Chitra , Arun, Anjali , Klausner, Jeffrey D , Reingold, Arthur L.	2007	The Epidemiology of Herpes Simplex Virus Type 2 Infection Among Married Women in Mysore, India	South Asia	HSV 2 seropositive was 11.3% (95%CI: 9.4%–13.6%).	Sexually Transmitted Diseases
Patzi Churqui, Marianela , Terrazas Aranda, Katty , Liljeqvist, Jan Åke , Lindh, Magnus , Eriksson, Kristina	2020	Prevalence of viral sexually transmittedinfections and HPV high risk genotypes in women in rural communities in theDepartment of La Paz, Bolivia	South America	The seroprevalence of HSV 2 was 53%	BMC Infectious Diseases
Kalu, E. I , Ojide, C. K , Fowotade, A , Nwadike, V. U.	2014	Sexual behavioral correlates with HSV 2 seroprevalence among pregnant women in Nigeria	West Africa	Seroprevalence of HSV 2 antibody was 47.3%	Journal of Infection in Developing Countries
Gomes Naveca, Felipe , Sabidó, Meritxell , Amaral Pires De Almeida, Tatiana , Araújo Veras, Elaine , Contreras Mejía , Matilde Del Carmen , Galban, Enrique , Benzaken, Adele Schwartz	2013	Etiology of Genital Ulcer Disease in a Sexually Transmitted Infection Reference Center in Manaus,Brazilian Amazon	South America	HSV 2 was detected in 55.3% of GUD samples,	PLoS ONE
Noda, Angel A , Blanco, Orestes , Correa, Consuelo , Pérez, Lissette , Kourí, Vivian , Rodríguez, Islay	2016	Etiology of Genital Ulcer Disease in Male Patients Attending a Sexually Transmitted Diseases Clinic: First Assessment in Cuba	Caribbean	The prevalence of HSV 2 was 51.3%	Sexually Transmitted Diseases

Miranda, C. A. N , Lima, É G , Lima, D. B. S. de , Cobucci, R. N. O , Cornetta, M. da C. de M , Fernandes, T. A. A. de M , Azevedo, P. R. M. de , Azevedo, J. C. V. de , Araújo, J. M. G. de , Fernandes, J. V.	2014	Genital Infection with Herpes Simplex Virus Types 1 and 2 in Women from Natal, Brazil	South America	For HSV 2, these rates were 12.3% for pregnant women and 15.5% non pregnant women	ISRN Obstetrics and Gynecology

HSV 2 Diagnostics

Authors	Year	Title	Geographical area of study population (according to UN classification)	Main findings/Prevalence	Journal
Gopal, R. , Gibbs, T., Slomka, M. J. , Whitworth, J. , Carpenter, L. M. , Vyse, A. , Brown, D. W. G.	2000	A monoclonal blocking EIA for herpes simplex virus type 2 antibody: validation for seroepidemiological studies in Africa	east africa	suitable test for epidemiological studies and type specific management, comparable sensetivity and specificity to western blot	Journal of Virological Methods
Haripriya, V. , Rajendran, P. , Thyagarajan, S. P. , Sripathy, M. S. , Parthiban, R. , Ram, C. C. M. , Gopalakrishnan, P. B. , Chandraseskhar, G. S. , Melani, Rajendran	2002	A study on the comparison between clinical and microbiological diagnoses of sexually transmitted diseases	South Asia	laboratory/microbiological shows better accuracy	Journal of the Indian Medical Association
Hogrefe, W., Su, X. , Song, J. , Ashley, R. , Kong, L.	2002	Detection of herpes simplex virus type 2 specific immunoglobulin G antibodies in African sera by using recombinant gG2, Western blotting, and gG2 inhibition	east africa and southern africa	comparable sensetivity and specificity	Journal of Clinical Microbiology
Liu, H., Detels, R., Yin, Y., Li, X., Visscher, B.	2003	Do STD clinics correctly diagnose STDs? An assessment of STD management in Hefei, China	east asia	poor accuracy of diagnostics HSV overdignosed	International Journal of STD & AIDS

Ndjoyi Mbiguino, A., Ozouaki, F. , Legoff, J. , Mbopi Kéou, F. X. , Si Mohamed, A. , Onas, I. N. , Avoune, E. , Bélec, L.	2003	Comparison of washing and swabbing procedures for collecting genital fluids to assess cervicovaginal shedding of herpes simplex virus type 2 DNA	central africa	cervicovaginal lavage/washing detects more than endocervicovaginal swabbing	Journal of Clinical Microbiology
Wang, QianQiu , Yang, Ping , Zhong, MingYing , Wang, GuangJu	2003	Validation of diagnostic algorithms for syndromic management of sexually transmitted diseases	east asia	syndromic approach not as specific and sensitive for diagnosing GUD (inc HSV 2) as for discharge STDs	Chinese Medical Journal (Beijing)
Dyck, E. van, Buvé, A. , Weiss, H. A. , Glynn, J. R. , Brown, D. W. G. , Deken, B. de , Parry, J. , Hayes, R. J.	2004	Performance of commercially available enzyme immunoassays for detection of antibodies against herpes simplex virus type 2 in African populations	east, central and west africa (sub saharan africa)	best 3 kits for african (sensitivity and specificity) sera were Gull Premier, Biokit and kalon biological. Herpeselect good in industrialsed countries but not africa, cannot assume results will be comparabl in different areas ofo the world.	Journal of Clinical Microbiology
Kara, P., Meric, B., Zeytinoglu, A., Ozsoz, M.	2004	Electrochemical DNA biosensor for the detection and discrimination of herpes simplex type I and type II viruses from PCR amplified real samples	western asia (turkey)	successful discrimination and detection, rapid and low cost, safer (no ethidium bromide)	Analytica Chimica Acta
Laeyendecker, O., Henson, C. , Gray, R. H. , Nguyen, R. H. N. , Horne, B. J. , Wawer, M. J. , Serwadda, D. , Kiwanuka, N. , Morrow, R. A. , Hogrefe, W. , Quinn, T. C.	2004	Performance of a commercial, type specific enzyme linked immunosorbent assay for detection of herpes simplex virus type 2 specific antibodies in Ugandans	east africa	HIV status did not effect performance of herpselect and does not explain different performance in different geo areas , difference may be due to different strains and gG2 epitope variations	Journal of Clinical Microbiology
Hoyo, C. , Hoffman, I. , Moser, B. K. , Hobbs, M. M. , Kazembe, P. , Krysiak, R. G. , Cohen, M. S.	2005	Improving the accuracy of syndromic diagnosis of genital ulcer disease in Malawi	southern africa	syndromic algorythm does not need to be weighted no longer therefore easier to use	Sexually Transmitted Diseases
Nagot, N. , Foulongne, V. , Becquart, P. , Mayaud, P. , Konate, I. , Ouedraogo, A. ,	2005	Longitudinal assessment of HIV 1 and HSV 2 shedding in the genital tract of West African women	west africa	eCVL is reliable method for HSV 2 detection, could be useful in future trials	JAIDS, Journal of Acquired Immune

Defer, M. C. , Weiss, H. , Perre, P. van de , Segondy, M.				with hiv and hsv positivity as an outcome	Deficiency Syndromes
Görander, Staffan , Mbwana, Judica , Lyamuya, Eligius , Lagergård, Teresa , Liljeqvist, Jan Ake	2006	Mature glycoprotein g presents high performance in diagnosing herpes simplex virus type 2 infection in sera of different tanzanian cohorts	east africa	all similar performance on high prevalence group but mgG2 ELISA performed best overall (accuracy) and most suitable for future studies and clinical practice. Higher sensetivity of hereselect in GUD pts that blood donors	Clinical and vaccine immunology : CVI
Legoff, Jérôme , Bouhlal, Hicham , Grésenguet, Gérard , Weiss, Helen , Khonde, Nzambi , Hocini, Hakim , Désiré, Nathalie , Si Mohamed, Ali , de Dieu Longo, Jean , Chemin, Cécile , Frost, Eric , Pépin, Jacques , Malkin, Jean Elie , Mayaud, Philippe , Bélec, Laurent	2006	Real time PCR quantification of genital shedding of herpes simplex virus (HSV) and human immunodeficiency virus (HIV) in women coinfected with HSV and HIV	west africa and central africa	real time PCR is useful and accurate for hsv 2 and is an affordable option for detecting shedding in future intervention studies	Journal of clinical microbiology
Li, Ding , Yang, Hao , Zhang, Wen Hong , Pan, Hao , Wen, Dong Qing , Han, Feng Chan , Guo, Hui Fang , Wang, Xiao Ming , Yan, Xiao Jun	2006	A simple parallel analytical method of prenatal screening	east asia	no sig difference between microarray and ELISA , microarray cheaper and easier therefore more suitable for mass prenatal screening, however early days and many problems need resolving	Gynecologic and obstetric investigation
Nascimento, M. C. , Ferreira, S. , Sabino, E. , Hamilton, I. , Parry, J. , Pannuti, C. S. , Mayaud, P.	2007	Performance of the HerpeSelect (Focus) and Kalon enzyme linked immunosorbent assays for detection of antibodies against herpes simplex virus type 2 by use of monoclonal antibody blocking enzyme immunoassay and clinicovirological reference standards in Brazil	South America	Kalon performed better than herpeselect in Brazil	Journal of Clinical Microbiology

Gamiel, J. L. , Tobian, A. A. R. , Laeyendecker, O. B. , Reynolds, S. J. , Morrow, R. A. , Serwadda, D. , Gray, R. H. , Quinn, T. C.	2008	Improved performance of enzyme linked immunosorbent assays and the effect of human immunodeficiency virus coinfection on the serologic detection of herpes simplex virus type 2 in Rakai, Uganda	east africa	kalon perfomed best in uganda, higher than standard cutoffs were requird to achieve optimum sensetivity and specificity though. Biokit point of care test had low specificity (56.1%). None of the assays affected by HIV status	Clinical and Vaccine Immunology
LeGoff, J. , Mayaud, P. , Gresenguet, G. , Weiss, H. A. , Nzambi, K. , Frost, E. , Pepin, J. , Belec, L.	2008	Performance of HerpeSelect and Kalon assays in detection of antibodies to herpes simplex virus type 2	west africa and central africa	Hereselect detects seroconversion more frequently and earlier than kalon. Hereselect more sensetive at detecting nonprimary first episodes than kalon. Early infection more difficult to detect with both assays and this may affect the true sensetivities of herepeselect on studies. HIV status did not affect performanceof either assay	Journal of Clinical Microbiology
Ngo, T. D. , Laeyendecker, O. , La, H. , Hogrefe, W. , Morrow, R. A. , Quinn, T. C.	2008	Use of commercial enzyme immunoassays to detect antibodies to the herpes simplex virus type 2 glycoprotein G in a low risk population in Hanoi, Vietnam	east asia	retesting herpeselect with biokit or kalon can significantly reduce % of false positive results	Clinical and Vaccine Immunology
Ngo, T. D. , Laeyendecker, O. , Morrow, R. A. , Lai, S. H. , Quinn, T. C.	2008	Comparison of three commercial immunoassays for detection of herpes simplex virus type 2 antibodies in commercial sex workers in Yunnan Province, China	east asia	herpeselect perfrmed the best very close to kalon, improved understanding of the performance of these assays in chinese sex workers	Clinical and Vaccine Immunology
Sen, P. , Sun, Y. J. , Tan, H. H. , Tan, S. H. , Chan, R.	2008	Comparison of nested polymerase chain reaction and virus culture for the diagnosis of genital herpes simplex virus infection	east asia	nested PCR much more accurate than culture. Can detect and type hsv th sufficient accuracy	

Yin, YuePing , Wu, ZunYou , Lin, C. Q. , Guan, JiHui , Wen, Y. , Li, L. , Detels, R. , Rotheram Borus, M. J.	2008	Syndromic and laboratory diagnosis of sexually transmitted infection: a comparative study in China	east asia	syndromic approach sensitivity was approximately 10%, syndromic approach not good for asymptomatic	International Journal of STD & AIDS
Shilpee, Choudhry , Ramachandran, V. G. , Shukla, Das , Bhattacharya, S. N. , Mogha, N. S.	2009	Serological profile of HSV 2 in patients attending STI clinic: evaluation of diagnostic utility of HSV 2 IgM detection	South Asia	serum IgM not valuable as a single test for genital herpes diagnosis, would only be used as a supportive method. It isnt useful in determining whether an infection was recently acquired	Indian Journal of Pathology & Microbiology
Smith, J. S. , Bailey, R. C. , Westreich, D. J. , Maclean, I. , Agot, K. , Ndinya Achola, J. O. , Hogrefe, W. , Morrow, R. A. , Moses, S.	2009	Herpes simplex virus type 2 antibody detection performance in Kisumu, Kenya, using the Herpeselect ELISA, Kalon ELISA, Western blot and inhibition testing	east africa	kalon performed better than herpeselect in young men in kenya	Sexually Transmitted Infections
Suntoke, T. R. , Hardick, A. , Tobian, A. A. R. , Mpoza, B. , Laeyendecker, O. , Serwadda, D. , Opendi, P. , Gaydos, C. A. , Gray, R. H. , Wawer, M. J. , Quinn, T. C. , Reynolds, S. J.	2009	Evaluation of multiplex real time PCR for detection of Haemophilus ducreyi, Treponema pallidum, herpes simplex virus type 1 and 2 in the diagnosis of genital ulcer disease in the Rakai District, Uganda	east africa	thisnmultiplex real time PCR is a sensetive and specific, rapid and reproducible way of detecting aetiology of GUD in resource poor areas, better performance than previous PCR studies in uganda	Sexually Transmitted Infections
Delany Moretlwe, S. , Jentsch, U. , Weiss, H. , Moyes, J. , Ashley Morrow, R. , Stevens, W. , Mayaud, P.	2010	Comparison of focus HerpesSelect and Kalon HSV 2 gG2 ELISA serological assays to detect herpes simplex virus type 2 antibodies in a South African population	southern africa	both these ELISA tests had low specificity in this south african population. Increasing inidec value of herpeselect fro 1.1 to 3.5 improved specificity. HIV seropositivity reduced herpeselect specificity by 30%. Alternative algorythm with improved sec and sensi is 2 tests (hepeselect then confirm with kalon.	Sexually Transmitted Infections

Lingappa, J. , Nakku Joloba, E. , Magaret, A. , Friedrich, D. , Dragavon, J. , Kambugu, F. , Joloba, M. , Whalen, C. , Coombs, R. , Celum, C. , Morrow, R. A.	2010	Sensitivity and specificity of herpes simplex virus 2 serological assays among HIV infected and uninfected urban Ugandans	east africa	best performance is by kalon alone or hereselect followed by biokit confirmatory testing. Had to increase cutoff index of herpeselect to achieve better specificity. HIV positivity decreased herepeselect specificity. Need more robust assays for HSV 2 and HIV public health activities in africa in point of care style	International Journal of STD & AIDS
Ng'ayo, M. O. , Friedrich, D. , Holmes, K. K. , Bukusi, E. , Morrow, R. A.	2010	Performance of HSV 2 type specific serological tests in men in Kenya	east africa	best performance was kalon using higher 1.5 cuoff. None of them perfect and need caution in interpretation. None of the rapid test suitable allone or as confirmatory test. Gen 2 no better than gen 1.	Journal of Virological Methods
Shilpee, Choudhry , Ramachandran, V. G. , Shukla, Das , Bhattacharya, S. N. , Mogha, N. S.	2010	Pattern of sexually transmitted infections and performance of syndromic management against etiological diagnosis in patients attending the sexually transmitted infection clinic of a tertiary care hospital	south asia	syndromic diagnosis 83% sensetive for HSV2 . Syndromic has its disadvantages and needs tobe regukarlyy reviewed and improved	Indian Journal of Sexually Transmitted Diseases and AIDS
LeGoff, Jérôme , Grésenguet, Gérard , Gody, Chrysostome , Longo, Jean De Dieu , Khonde, Nzambi , Weiss, Helen A. , Mayaud, Philippe , Bélec, Laurent	2011	Performance of the BioPlex 2200 multiplexing immunoassay platform for the detection of herpes simplex virus type 2 specific antibodies in African settings	west africa and central africa (CAR and Ghana)	excellent agreement between bioplex and hereselect at all cutoffs. Bioples has advantage of detecting hsv 1 and 2 so should be considered for future cliical and epidemiological uses	Clinical and vaccine immunology : CVI
Muvunyi, C. M. , Dhont, N. , Verhelst, R. , Crucitti, T. , Reijans, M. , Mulders, B. ,	2011	Evaluation of a new multiplex polymerase chain reaction assay STDFinder for the simultaneous	east africa	STD Finder multples PCR performs as well as	Diagnostic Microbiology and Infectious Disease

Simons, G. , Temmerman, M. , Claeys, G. , Padalko, E.		detection of 7 sexually transmitted disease pathogens		single/mono PCR assays. Multiplexing reduced cost	
Neal, J. D. , Tobian, A. A. , Laeyendecker, O. , Ngo, T. D. , Redd, A. D. , Reynolds, S. J. , Morrow, R. A. , Manucci, J. L. , Serwadda, D. , Gray, R. H. , Quinn, T. C. , Neal, J. D. , Tobian, A. A. R. , Laeyendecker, O. , Ngo, T. D. , Redd, A. D. , Reynolds, S. J. , Ashley Morrow, R. , Manucci, J. L. , Serwadda, D.	2011	Performance of the Euroline Western blot assay in the detection of herpes simplex virus type 2 antibody in Uganda, China and the USA	east africa and east asia	consistently high sensetivity of euroline in china and uganda but specificity a lot lower (and also lower than kalon and hereselect) in uganda. Euroline is commercially available and easy to perform	International Journal of STD & AIDS
Ranu, H. , Lee, J. , Chio, M. , Sen, P.	2011	Tumour like presentations of anogenital herpes simplex in HIV positive patients	east asia	HSV 2 should be considered in nodular angogenital erosions in HIV positive people , PCR should be carried out as culture may be negative. Highlight urgent need for intervwntions to targt this syndemic relationship	International Journal of STD & AIDS
CanchéPech, J. R. , González Losa, M. del R. , Ayora Talavera, G. , Carrillo Martinez, J. , Sáenz Carbonell, L. A. , Córdova Lara, I. , Conde Ferráez, L.	2012	Herpes simplex virus infection in obstetric patients: comparing serology vs. real time PCR	South America	IgM did not correlate to genital tract shedding. So presence genital viral DNA (PCR) is better for identifying mothers at risk	
Tada, D. G. , Neeta, Khandelwal	2012	Serum HSV 1 and 2 IgM in patients of sexually transmitted diseases	South asia	hsv 2 igm could be used as a supportive diagnostic test for genital herpes and for survellance activities however, overall not hat helpful in knowing when the infection was acquired	Annals of Tropical Medicine and Public Health
Kapil, Goyal , Ratho, R. K. , Kanwar, A. J. , Baijayantimala, Mishra , Singh, M. P.	2013	Role of polymerase chain reaction in detection of genital herpes	south asia	PCR performed much better than tzanck smears, antigen detection and culture. 7 additional cases detected with PCR than	Indian Journal of Dermatology, Venereology and Leprology

				with all other direct methods. Authors suggest that larger prospective studies should be carried outin order o replace viral culture with PCR as gold standard. advantages ove rculture in genital herpes: can detect subclinical shedding and undoagnosed symptomatic ulcers	
Rao, G. , Das, A. , Prabhakar, P. , Nema, V. , Risbud, A. R.	2013	Alteration in sample preparation to increase the yield of multiplex Polymerase Chain Reaction assay for diagnosis of genital ulcer disease	south asia	phenol chloroform extraction and ethanol precipitation helped eliminate PCR inhibitors and increase detected cases, but it is a long process and therefore not suitable for routine work, maybe for negative samples only	Indian Journal of Medical Microbiology
Rodrigues, D. , de Paris, F. , Paiva, R. M.	2013	Minimum detection limit of an in house nested PCR assay for herpes simplex virus and varicella zoster virus	South America	similar detection limit to previous studies	Revista da Sociedade Brasileira de Medicina Tropical
Tankhiwale, S. S. , Chavan, S. P.	2013	Comparative study of syndromic and etiological diagnosis of sexually transmitted infection except human immunodeficiency virus in sexually transmitted infection and reproductive tract infection clinic attendees in central India	South Asia	confirmed that syndromic ha disadvantages and highlights the need to review its relevance regularly depending on geographical area and population and STI trends in that area	International Journal of Medicine and Public Health
Amirjannati, N. , Yaghmaei, F. , Akhondi, M. M. , Nasiri, M. , Heidari Vala, H. , Sehhat, Z.	2014	Molecular and serologic diagnostic approaches; the prevalence of herpes simplex in idiopathic men infertile	south asia	PCR is prefered over serology	Iranian Journal of Reproductive Medicine
Banihashemi, M. , Pezeshkpoor, F. ,	2014	Tzanck smears in herpes simplex virus infections	South Asia	tzanck not very specific ad has limitations but is very	Iranian Journal of Dermatology

Yousefzadeh, H. , Livani, F. , Ghandeharian, G.				cost effective therefore may be used when other methods are unavailable in resource poor settings	
Gimenes, F. , Medina, F. S. , Abreu, A. L. P. de , Irie, M. M. T. , Esquiçati, I. B. , Malagutti, N. , Vasconcellos, V. R. B. , Discacciati, M. G. , Bonini, M. G. , Maria Engler, S. S. , Consolaro, M. E. L.	2014	Sensitive simultaneous detection of seven sexually transmitted agents in semen by multiplex PCR and of HPV by single PCR	South America	M PCR is an accurate and useful tool for detecting HSV 2 nd other STDs in sperm	PLoS ONE
Hong, YunJi , Lim, MiSuk , Hwang, SangMee , Kim, TaekSoo , Park, KyoungUn , Song, JungHan , Kim, EuiChong	2014	Detection of herpes simplex and varicella zoster virus in clinical specimens by multiplex real time PCR and melting curve analysis	east asia	accurate and suitable method for detecting and typing HSV infection, could replace culture	BioMed Research International
Otieno, F. O. , Ndivo, R. , Oswago, S. , Ondiek, J. , Pals, S. , McLellan Lemal, E. , Chen, R. T. , Chege, W. , Gray, K. M.	2014	Evaluation of syndromic management of sexually transmitted infections within the Kisumu Incidence Cohort Study	east africa	syndromic diagnosis of sti does not perform sufficiently well in Kismu, Kenya. Need more development of affordable cost effective laboratory methods	International Journal of STD & AIDS
Ryan, C. E. , Simbiken, C. S. , Agius, P. A. , Allen, J. , Sauk, J. , Kaima, P. , Kombati, Z. , Siba, P. , Kaldor, J. M. , Vallely, A.	2014	Comparative performance of the Kalon and HerpeSelect enzyme linked immunosorbant assays to determine the prevalence of herpes simplex virus type 2 in Papua New Guinea	east asia	herpeselect lacked specificity (many false positives likely) and terefore recommended kalon for future studies in PNG	Sexual Health
Shah, N. S. , Kim, E. , Ayala, F. de M. H. , Guardado Escobar, M. E. , Nieto, A. I. , Kim, A. A. , Paz Bailey, G.	2014	Performance and comparison of self reported STI symptoms among high risk populations MSM, sex workers, persons living with HIV/AIDS in El Salvador	central america	self reeported symptoms not a good metho for high risk people in el salvadoor, however due to poor lab resources and high prevalence of genital herpes perhaps overdiagnosis through synromic management would be acceptable, hoever, nt good for viral	International Journal of STD & AIDS

				resistance. Need some sort of screening for high risk people in E.Sal	
Wu, DongSheng , Wu, YuanJian , Wang, LiuHong , Xu, WeiDong , Zhong, Qiao	2014	Evaluation of a novel array based toxoplasma, rubella, cytomegalovirus, and herpes simplex virus IgG enzyme linked immunosorbent assay and its comparison with virion/serion enzyme linked immunosorbent assays	east asia	BGI ELISA showed near perfect agreement with conventional ELISA and is a suitable mthod for clinical practice	Annals of Laboratory Medicine
Baetselier, I. de , Menten, J. , Cuylaerts, V. , Ahmed, K. , Deese, J. , Damme, L. van , Crucitti, T.	2015	Prevalence and incidence estimation of HSV 2 by two IgG ELISA methods among South African women at high risk of HIV	southern africa	kalon and hereselect similar performance (concordant). Kalon index reference djusted to 0.66 to catch early infections without losing specificity	PLoS ONE
Kalu, E. I. , Ojide, C. K. , Nwadike, V. U. , Ogbaini Emovon, E. , Okafor, G. O. C.	2015	Validity of genito urinary discharges, genital ulcers and genital rashes as indicators of seroincident HSV 2 infection	west africa	genito urinary discharges, genital ulcers and genital rashes are valis as a screening tool in porr resourced settings to suggest incident genital herpes	Asian Pacific Journal of Tropical Disease
Lamers, Susanna L. , Newman, Ruchi M. , Laeyendecker, Oliver , Tobian, Aaron A. R. , Colgrove, Robert C. , Ray, Stuart C. , Koelle, David M. , Cohen, Jeffrey , Knipe, David M. , Quinn, Thomas C.	2015	Global Diversity within and between Human Herpesvirus 1 and 2 Glycoproteins	west africa, southern africa and asia	glycoprotein g showed variation across all the different locations, this will help refine performance of diagnostic test. Hsv2 gG less diverse than 1	Journal of virology
Patel, E. U. , Manucci, J. , Kahle, E. M. , Lingappa, J. R. , Morrow, R. A. , Piwowar Manning, E. , James, A. , Maluzi, K. F. , Cheeba, M. M. , Gray, G. , Delany Moretlwe, S. , Inambao, M. , Vwalika, B. , Quinn, T. C. , Laeyendecker, O.	2015	Precision of the Kalon Herpes Simplex Virus type 2 IgG ELISA: an international inter laboratory assessment	Southern africa	high precision and reproducibility in different labs in southern african countries	BMC Infectious Diseases

Li, ZhiYan , Yan, Rong , Yan, CunLing , Liu, Ping , Feng, ZhenRu	2016	Evaluation of an automated chemiluminescent immunoassay in typing detection of IgG antibodies against herpes simplex virus	east asia	CLIA is plausible method of detecting hsv 2 gG	Journal of Clinical Laboratory Analysis
Nsobya, S. L. , Hewett, P. C. , Kalibala, S. , Mensch, B. S.	2016	Performance of Kalon herpes simplex virus 2 assay using dried blood spots among young women in Uganda	east africa	DBS with kalon had poor accuracy performance	African Journal of Laboratory Medicine
Vrushali, Patwardhan , Preena, Bhalla	2016	Role of type specific herpes simplex virus 1 and 2 serology as a diagnostic modality in patients with clinically suspected genital herpes: a comparative study in Indian population from a tertiary care hospital	south asia	ELISA had better sensetivity and specificity in recurrent genital erpes episodes than in primary episodes. Could be suitable for use in supporting a diagnosis of recurrent genital ulcers	Indian Journal of Pathology & Microbiology
Yang, JinFang , Zhao, ChangZhen , Lu, KeXin	2016	Development and application of a rapid detection system for human papillomavirus and Herpes simplex virus 2 by loop mediated isothermal amplification assay	east asia	accurate enough to be used as part of extensive screening of hsv 2 since it is ver cost efefctive	Microbial Pathogenesis
Hobbs, M. M. , Mwanyumba, S. W. , Luseno, W. K. , Hartman, S. , Halpern, C. T. , Hallfors, D. D. , Cho, HyunSan	2017	Evaluation of herpes simplex virus type 2 serological tests for use with dried blood spots in Kenya	east africa	kalon performance was poor, herpeselect has sen and specs of 98.8 and 98.9 . Suitable and valuable in low resource settings dur to convenience and utility of DBS compared to storing sera	Sexually Transmitted Diseases
Lima, L. R. P. , Silva, A. P. da , Schmidt Chanasit, J. , Paula, V. S. de	2017	Diagnosis of human herpes virus 1 and 2 (HHV 1 and HHV 2): use of a synthetic standard curve for absolute quantification by real time polymerase chain reaction	south america	" This study demonstrated that synthetic curves could be used as alternative standard curves for HHV diagnosis, since they showed similar re sults when compared to viral DNA curves."	Memórias do Instituto Oswaldo Cruz

Fettahlıoğlu Karaman, Bilge	2018	The role of Tzanck smear in the diagnosis of herpetic lesions older than three days	western asia (turkey)	tzanck smears still useful after 3 days especially if there are still vesicles	Cukurova University, 2018.
Finger Jardim, F. , Avila, E. C. , Hora, V. P. da , Santos, P. C. dos , Gonçalves, C. V. , Mor, G. , Martinez, A. M. B. de , Soares, M. A.	2018	Herpes simplex virus type 2 IgG antibodies in sera of umbilical cord as a proxy for placental infection in asymptomatic pregnant women	South America	circulating IgG can be indicative of placental infection in asymptomati women	American Journal of Reproductive Immunology
Kaida, A. , Dietrich, J. J. , Laher, F. , Beksinska, M. , Jaggernath, M. , Bardsley, M. , Smith, P. , Cotton, L. , Chitneni, P. , Closson, K. , Lewis, D. A. , Smit, J. A. , Ndung'u, T. , Brockman, M. , Gray, G.	2018	A high baurden of asymptomatic genital tract infections undermines the syndromic management approach among adolescents and young adults in South Africa: implications for HIV prevention efforts	Southern africa	syndromic diagnosis not sensetive enough in youths in SA and leaves many symptomatic patients at risk of HIV acquisition	BMC Infectious Diseases
Lee, ChunKiat , Chai, CheanNee , Capinpin, S. M. , Ang, A. , Ng, SauYoke , Lee, PeakLing , Ng, WaiSiong , Yan, G. , Lee, HongKai , Chiu, L. L. , Jureen, R. , Yan, B. , Loh, TzePing	2018	Evaluation of the Luminex ARIES HSV 1&2 assay and comparison with the FTD Neuro 9 and in house real time PCR assays for detecting herpes simplex viruses	east asia	ARIES is a valuable alternative for detecting HSV 2 in genital infection, particularly rapid method. Low mainenance and calibration requirements	Annals of Laboratory Medicine
Nasrallah, G. K., Dargham, S. R., Sahara, A. S. , Elsidiq, M. S. , Abu Raddad, L. J.	2019	Performance of four diagnostic assays for detecting herpes simplex virus type 2 antibodies in the Middle East and North Africa	north africa and western asia/middle east	good overall concordance ofall assays, however caution should be used using any of these alone in a epi study and a 2 test algorythm is suggested to improve sensetivity and specificity	Journal of Clinical Virology
Rodrigues, L. L. S. , Pilotto, J. H. , Lima, L. R. P. , Gaydos, C. A. , Hardick, J. , Morgado, M. G. , Martinelli, K. G. , Paula, V. S. de , Nicol, A. F.	2019	Self collected versus clinician collected samples for HSV 2 and HSV 2/HPV screening in HIV infected and uninfected women in the Tapajós region, Amazon, Brazil	South America	slef collection as good as clinician and could save time and resources	International Journal of STD & AIDS
Chakraborty, Payal , Norris, Alison H. , Huber Krum, Sarah , Garver, Sarah , Hood, Robert B. , Banda, Venson , Esber,	2020	An Assessment of Risk Factors for Herpes Simplex Virus Type 2 Infection in Malawian Women	southeren africa (malawi)	diffferent risk facors associated with the diffrerent cutpoints need to be aware of which	Sexually Transmitted Diseases

Allahna , Patricia, Carr Reese , Krysiak, Robert , Turner, Abigail Norris		Using 2 Classifications for the HerpeSelect 2 Test		cutpount when recruiting 'high risk' people since the characteristics will be different	
