# Supplementary material for: Twenty years of herpes simplex virus type 2 (HSV-2) research in low-income and middle-income countries: systematic evaluation of progress made in addressing WHO prioritiesfor research in HSV-2 epidemiology and diagnostics
Source: BMJ Glob Health. 2024 Jul 4;9(7):e012717. doi: 10.1136/bmjgh-2023-012717 (PMC11227754; doi:10.1136/bmjgh-2023-012717)
Supplement: Supplementary data [file bmjgh-2023-012717supp003.pdf]

Appendix 3 - Reasons for exclusion after full-text eligibility review for each research area

HSV-2 Epidemiology

Number of excluded records: 116

Reasons for exclusions: Studies on HIV and HSV-2 interaction (n=17), STI prevalence without HSV-2 data (n=19), based on High Income Countries (n=13), studies on HSV-1 Epidemiology (n=15), studies on other aspects of HSV-2 without HSV-2 epidemiology data (n=37), Duplicate records (n=9) and research protocols and letters (n=6)

HSV-2 Diagnostic

Number of excluded records: 30

Reasons for exclusions: focussed on diagnosing HSV-2 meningitis or keratitis (n=17), based on High-Income Countries (n=9), and studies on HSV-1 diagnosis (n=4)
