# Supplementary material for: Twenty years of herpes simplex virus type 2 (HSV-2) research in low-income and middle-income countries: systematic evaluation of progress made in addressing WHO prioritiesfor research in HSV-2 epidemiology and diagnostics
Source: BMJ Glob Health. 2024 Jul 4;9(7):e012717. doi: 10.1136/bmjgh-2023-012717 (PMC11227754; doi:10.1136/bmjgh-2023-012717)
Supplement: Supplementary data [file bmjgh-2023-012717supp004.pdf]

Reflexivity Statement

A REVIEW OF AUTHORSHIP IN HERPES SIMPLEX VIRUS TYPE-2 (HSV-2) RESEARCH CONDUCTED IN LOW AND MIDDLE-INCOME COUNTRIES BETWEEN 2000 AND 2020.

Belinder Naha, Ela Owen, Muna Jama, Angela Obasi, Emily Clarke

Context

This research reviewed authorship within a specific research field using publicly available searchable documents. Because it was commissioned and conducted in a high-income country (HIC) and did not directly involve human participants in, or primary data from, low or middle income countries, it does not fall into the category of publication that usually requires reflexivity statements. However, because the focus of this work and its companion papers is the publication of research conducted in low- and middle-income countries (LMIC) we felt it important to reflect on the issues within the reflexivity statement guidance, both for this paper and its two companions.

How does this study address local research and policy priorities?

The inequitable distribution of research authorship between HIC and LMIC contributors in research that is conducted in LMIC is increasingly recognised as a priority within global health research. The importance of inequities in gender are also being recognised. Documentation and analysis of these inequities are key to developing effective steps to address them.

How were local researchers involved in study design?

We acknowledge the overrepresentation of HIC researchers within the research team. As stated above, the study was commissioned and conducted in a HIC. However, two of the authors bring local perspectives through country of birth, nationality (MJ) and prolonged LMIC residence (MJ and AO). AO (joint senor author) is a HIC researcher with experience in international research collaborations with LMIC researchers. Although based in a HIC, she has a particular interest in promoting more equitable recognition of groups that are currently disadvantaged within the ecosystem and is actively engaged in collaborations to address this.[i] AO was also an early career researcher who attended the original WHO workshop while based in an LMIC.

How has funding been used to support the local research team?

No funding was required.

How are the research staff who conducted data collection and data interpretation acknowledged?

All authors contributed to instrument design and data acquisition. BN & EO led the analysis and produced the first draft of the manuscript. This has been acknowledged through their joint first authorship.

How have members of the research partnership been provided with access to study data?

All members have access to the study data.

How were data used to develop analytical and writing skills within the partnership?

BN, EO and MJ are early career researchers. They were supported to lead the analysis and in writing through regular meetings and reviewing of drafts with and by AO and EC. This has included support

to attend conferences where they have presented their work, and to act as first and/or corresponding authors for the companion papers (MJ and EO).

How were research partners supported to develop writing skills?
There were no LMIC based partners at the start of this work, although MJ has now returned to Somalia. As stated above, she is a first and corresponding author on a companion paper.

How will research products be shared to address local needs?
This research will be published as open access and disseminated through social media

How is the leadership, contribution and ownership of this work by LMIC researchers recognised within the authorship?
As stated above, this work has been conducted by a largely HIC team but MJ who is based in Somalia is the first and corresponding author on a companion paper

How have early career researchers across the partnership been included within the authorship team?
Early career researchers (BN, EO, MJ) have been involved in data analysis and writing of the manuscripts.

How had gender balance been addressed within the authorship?
All authors (BN, EO, MJ, EC, AO) are female. We acknowledge the lack of male researchers within the research team.

How has the project contributed to training of LMIC researchers?
The training in analysis and writing that MJ has received as part of this work will be of value in her research and career in Somalia.

How has the project contributed to improvements in local infrastructure?
This project has not directly contributed to improvements in local infrastructure.

What safeguarding procedures were used to protect local study participants and researchers?
There was no primary data collection within this research.

---

[i] Morton, B., Vercueil, A., Masekela, R., Heinz, E., Reimer, L., Saleh, S., Kalinga, C., Seekles, M., Biccard, B., Chakaya, J., Abimbola, S., Obasi, A. and Oriyo, N. (2022), Consensus statement on measures to promote equitable authorship in the publication of research from international partnerships. Anaesthesia, 77: 264-276. https://doi.org/10.1111/anae.15597
